# Genomic and Transcriptomic Insights Into the Autotrophic Metabolism on H_2_ + CO_2_ or CO of the Thermophilic Acetogenic Model Organism *Moorella thermoacetica*

**DOI:** 10.1111/1462-2920.70289

**Published:** 2026-04-01

**Authors:** Florian P. Rosenbaum, Anja Poehlein, Rolf Daniel, Volker Müller

**Affiliations:** ^1^ Department of Molecular Microbiology & Bioenergetics, Institute of Molecular Biosciences Johann Wolfgang Goethe University Frankfurt Germany; ^2^ Genomic and Applied Microbiology & Göttingen Genomics Laboratory Georg‐August University Göttingen Göttingen Germany

**Keywords:** [NiFe]‐hydrogenase, acetogenesis, carbon monoxide, formate cycle, formate hydrogenlyase, hydrogen, NADH dehydrogenase

## Abstract

Although the Wood‐Ljungdahl pathway, a wide‐spread pathway for CO_2_ fixation in anaerobic microorganisms, was elucidated in the thermophilic acetogenic bacterium *Moorella thermoacetica* , still relatively little is known about the enzymes involved in hydrogen oxidation, CO_2_ fixation, energy conservation and the role of quinones and cytochromes. Here, we have used transcriptomics, enzyme assays and genome analyses to identify missing links. NADPH, generated by a [FeFe] hydrogenase, is the reductant for CO_2_ reduction to formate, a key reaction in CO_2_ fixation. This reaction is slightly endergonic under standard conditions but becomes thermodynamically feasible at high environmental H_2_ concentrations. In addition, formate is taken out of equilibrium by a formate dehydrogenase that potentially forms a complex with an energy‐converting hydrogenase (Fdh–Ech), a novel respiratory enzyme in acetogens. Under low H_2_ concentrations, the complex can drive the reverse, endergonic reaction. In addition, we postulate a formate cycle involving a periplasmic, cytochrome *b*‐containing formate dehydrogenase. A NADH dehydrogenase‐like enzyme that uses reduced ferredoxin instead of NADH to reduce menaquinone is also involved in energy conservation. The data are summarised in a comprehensive metabolic and bioenergetic model of acetogenesis from H_2_ + CO_2_ and CO in *M. thermoacetica* .

## Graphical abstract

*Moorella thermoacetica* is the model organism for elucidation of acetogenesis. The reduction of CO_2_ to acetate is well studied, but how redox carrier balancing and energy conservation are maintained during autotrophic growth and how H_2_ is oxidised remains enigmatic. We addressed those questions using physiological, biochemical and bioinformatical approaches.

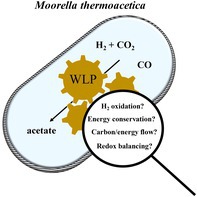

## 1 Introduction

Acetogenic bacteria are a specialised group of strictly anaerobic bacteria that use the Wood‐Ljungdahl pathway (WLP) for CO_2_ fixation as their central metabolic pathway (Drake et al. 2008). CO_2_ is reduced to acetate with electrons derived from organic or inorganic electron donors (Drake et al. 2008; Schuchmann and Müller 2016). Briefly, the WLP has two branches, the methyl and the carbonyl branch (Ljungdahl 1986). In the methyl branch, CO_2_ is first reduced to formate. This reaction is catalysed by formate dehydrogenases/CO_2_ reductases and is the key step in CO_2_ fixation but it also presents the first energetic hurdle in the pathway. The redox potential of the CO_2_/formate couple is rather low (*E*_0_′ = −432 mV). Therefore, NADH (*E*_0_′ = −320 mV; *E*′ = −270 mV) (Thauer et al. 1977) cannot drive CO_2_ reduction to formate. The standard redox potential of NADPH is the same as for NADH but its cellular potential (*E*′ = −370 mV) is in the same magnitude as that of the CO_2_/formate couple; CO_2_ reduction with NADPH is close to equilibrium and small changes in the concentrations of the reactants can make CO_2_ reduction exergonic or endergonic. NADPH‐dependent formate dehydrogenases engaged in CO_2_ reduction are known in acetogens (Yamamoto et al. 1983). Reduced ferredoxin (*E*_0_′ = −450 − −500 mV) has a redox potential low enough to drive CO_2_ reduction. In addition, electron confurcating formate dehydrogenases are present in acetogens that use a low potential co‐reductant such as ferredoxin to drive endergonic electron transfer from NADH to CO_2_ (Wang, Huang, Kahnt, Müller, et al. 2013; Wang, Huang, Kahnt, and Thauer 2013; Dietrich et al. 2021).

Formate is then bound to the C1 carrier tetrahydrofolate (THF) at the expense of one ATP (Sun et al. 1969; Ljungdahl et al. 1970). Next, water is split off from formyl‐THF and methenyl‐THF is formed (Clark and Ljungdahl 1982). Methenyl‐THF is further reduced to methylene‐THF (Clark and Ljungdahl 1982) and methyl‐THF. There are four different types of methylene‐THF reductases known in acetogens (Öppinger et al. 2022); Types I and III use NADH as reductant, Type II ferredoxin and Type IV most likely use electron bifurcation/confurcation and NADH and methylene‐THF as an electron carrier, but the third one is unknown (Mock et al. 2014). The methyl group is then transferred to a corrinoid‐iron sulphur‐protein (CoFeSP) (Drake et al. 1981). In the carbonyl branch, CO_2_ is reduced to enzyme‐bound CO with reduced ferredoxin as reductant (Ragsdale et al. 1983; Seravalli et al. 1997). The key enzyme of the pathway, the CO dehydrogenase/acetyl‐CoA synthase (CODH/ACS) catalyses the condensation of methyl‐CoFeSP and enzyme‐bound CO as well as coenzyme A (CoA), resulting in acetyl‐CoA that is further converted to acetate by a phosphotransacetylase and acetate kinase, yielding one ATP (Schaupp and Ljungdahl 1974; Drake et al. 1981). CO_2_ reduction to acetyl‐CoA requires one ATP whereas acetogenesis from H_2_ + CO_2_ is energy neutral (Wood et al. 1986; Ljungdahl 1994).

Additional chemiosmotic mechanisms of ATP synthesis are present, that allow for growth on H_2_ + CO_2_. To establish the transmembrane electrochemical ion gradient acetogenic bacteria studied so far either possess the ferredoxin:NAD oxidoreductase (Rnf complex) or the ferredoxin:H^+^ oxidoreductase (energy converting hydrogenase, Ech) as respiratory complexes (Rosenbaum and Müller 2021). These enzymes are mutually exclusive and every acetogen studied so far either has Rnf or Ech and both complexes do neither have cytochromes nor quinones (Rosenbaum and Müller 2021). Rnf as well as Ech are bidirectional and can go either way, depending on the cellular conditions. For example, the Rnf complex works to reduce NAD^+^ under autotrophic conditions, but to reduce ferredoxin with NADH as reductant during growth on low energy substrates such as methanol or ethanol (Bertsch et al. 2016; Kremp et al. 2018; Westphal et al. 2018). Interestingly, cytochromes and quinones are found in addition to either Rnf or Ech in a minority of acetogens (Rosenbaum and Müller 2021). The role of cytochromes or quinones in energy conservation in acetogens is still enigmatic. *Sporomusa* strains are known to have a membrane‐bound, cytochrome *b*‐containing hydrogenase and recently we found an electron transport from hydrogen to methyl‐THF in *Sporomusa ovata* which represents a third way of energy conservation in acetogens (Kremp et al. 2022). Cytochromes have also recently been shown to be involved in the reduction of alternative electron acceptors such nitrate in *S. ovata* (Waschinger et al. 2026).

The thermophilic acetogen *Moorella thermoacetica* (formerly *Clostridium thermoaceticum*) has an outstanding role in the group of acetogenic bacteria; cytochromes and quinones were discovered there for the first time in acetogens and it served as a model organism for the elucidation of the WLP (Wood et al. 1986). Despite its outstanding position in the group of acetogenic bacteria, yet little is known about the enzymes involved in hydrogen oxidation, CO_2_ fixation, energy conservation and the role of quinones and cytochromes. Here we provide experimental data such as growth experiments, genome‐wide transcription and bioinformatic analyses as well as genome analyses that shed new light on autotrophic growth of *M. thermoacetica* on H_2_ + CO_2_ or CO and its bioenergetics.

## 2 Results

### 2.1 Genome‐Wide Expression Profiling of Cells Growing on H_2_ + CO_2_ or CO

**FIGURE 1:**
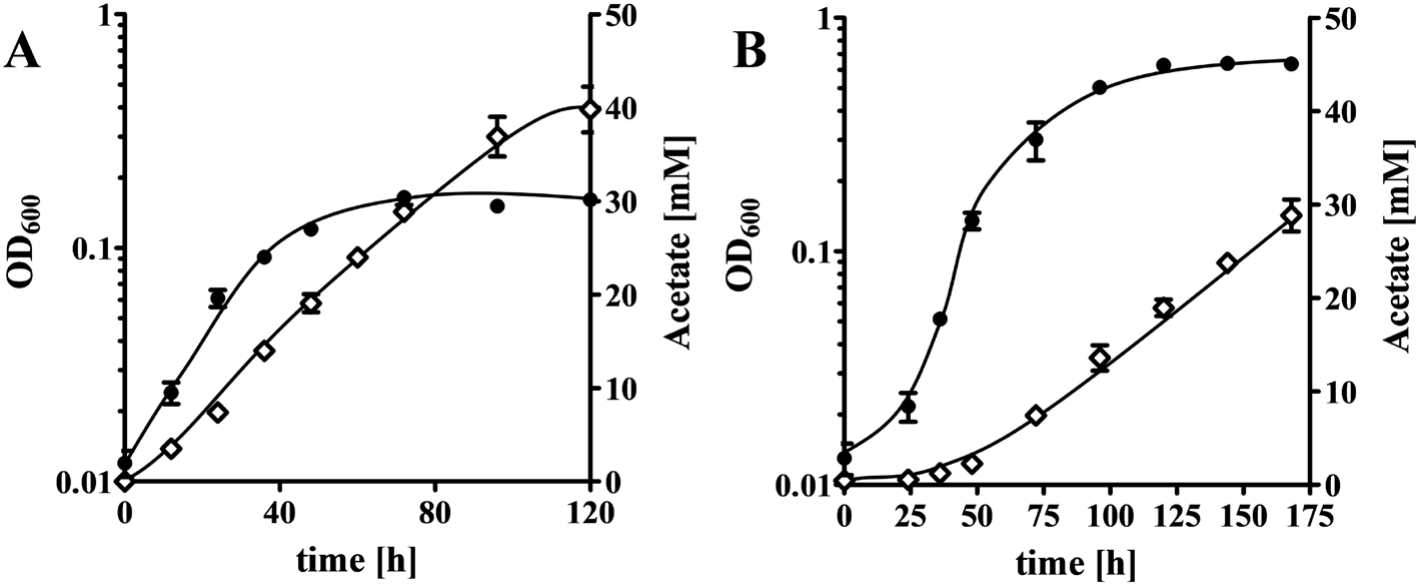
Growth of *Moorella thermoacetica* on H_2_ + CO_2_ (A) or CO (B). Cells were grown at 55°C in bicarbonate‐buffered medium under either a H_2_ + CO_2_ (80/20 [v/v]) or CO (100%) atmosphere. Optical density was monitored at 600 nm (OD, ●) and acetate (◊) concentrations were measured by HPLC (*n* = 3; SD).

**FIGURE 2:**
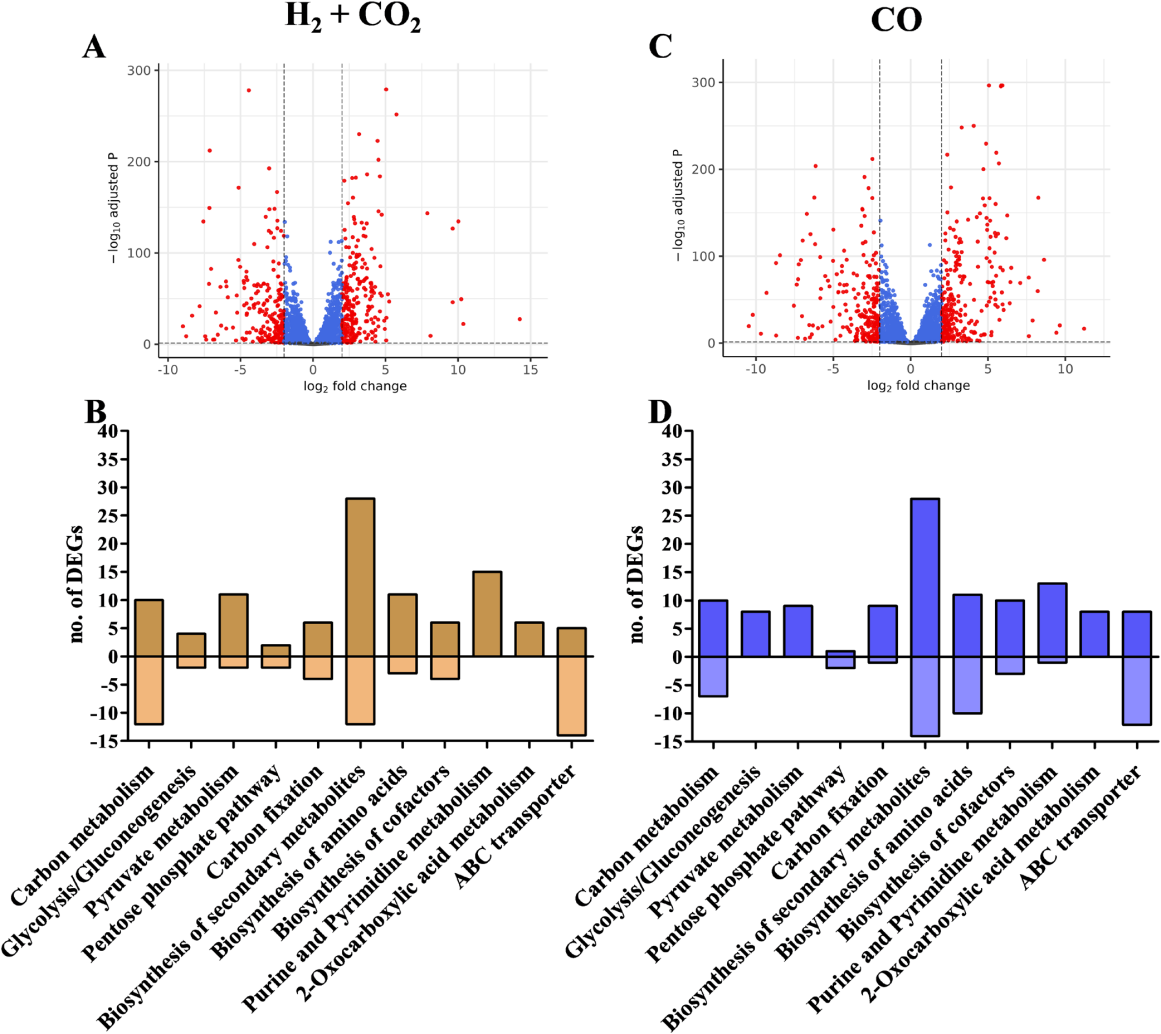
Vulcano plots and KEGG categorisation of DEGs of autotrophically grown *Moorella thermoacetica*. Vulcano plots of cells grown on H_2_ + CO_2_ (A) or CO (C), depicting all 2594 protein encoding genes; a log2FC of + 2/−2 and a *p*‐adjust value > 0.05 was used as threshold. Red, genes meeting these criteria (DEGs); blue, genes meeting the *p*‐adjust value; grey, genes with no significant differential expression. Number of DEGs annotated in selected KEGG pathways of cells grown on H_2_ + CO_2_ (B) or CO (D).

For the transcriptome analyses, cells had to be adapted to H_2_ + CO_2_ and CO. First, cells were grown on H_2_ + CO_2_ for several transfers. After three transfers, the culture was then used for further adaptation on CO. With H_2_ + CO_2_ as substrate the culture reached a final OD of 0.16 ± 0.01 and the doubling time was 12.4 h (growth rate (*μ*) = 0.055 h^−1^). Cultures growing on CO reached a final OD of 0.64 ± 0.01 with a doubling time of 9.2 h (*μ* = 0.075 h^−1^) (Figure 1). Acetate was the only product formed under both growth conditions. For transcriptome analyses, samples were taken during the exponential growth phase, RNA was isolated, transcribed into cDNA and quantified. Using a log_2_‐fold change (log2FC) of +2/−2 and a *p*‐adjust value of < 0.05 as threshold, a total of 489 genes of all 2594 genes were considered as differentially expressed genes (DEGs) (18.85%) in cells grown on H_2_ + CO_2_ and 517 genes (19.93%) in cells grown on CO (Figure 2A,C; Tables S1–S4). To give an overview of their distribution the DEGs are sorted according to their role in metabolic processes based on KEGG annotation. The metabolic processes affected mostly in H_2_ + CO_2_‐grown cells were the biosynthesis of secondary metabolites (30 DEGs), carbon metabolism (22 DEGs), purine and pyrimidine metabolism (15 DEGs), pyruvate metabolism (13 DEGs) and biosynthesis of amino acids (14 DEGs) (Figure 2B). In CO‐grown cells, the biosynthesis of secondary metabolites (32 DEGs), biosynthesis of amino acids (21 DEGs), carbon metabolism (17 DEGs), purine and pyrimidine metabolism (14 DEGs) and biosynthesis of cofactors (14 DEGs) were mostly affected (Figure 2D). In addition, 19 and 20 DEGs encoding ABC‐transporter were found in the transcriptome of H_2_ + CO_2_‐ or CO‐grown cells, respectively. We focussed our further analyses on genes encoding proteins involved in the WLP, redox carrier balancing and energy conservation.

### 2.2 DEGs Related to the WLP, Energy Conservation and Redox Balancing in H_2_ + CO_2_ ‐Grown Cells

The change from heterotrophic to autotrophic growth requires primarily changes in substrate oxidation, redox carrier balancing and energy conservation, since the WLP remains to be active under both conditions. Nonetheless the transcript levels of genes encoding the methyltransferase and acetate kinase genes were increased by a log2FC of +2.0 when cells grew on H_2_ + CO_2_ (Figure S1). All other genes encoding enzymes of the WLP were not differentially expressed. The transcript levels of the gene encoding the monofunctional CODH were reduced (log2FC of −4.1; Table S2) but the transcript count for the CODH/ACS encoding genes went up from 18,000 to 53,000 counts which explains the 6‐fold higher CO‐dependent ferredoxin reductase activity (Table 1). The genome of *M. thermoacetica* encodes two soluble hydrogenases, the electron‐bifurcating hydrogenase and the NADP^+^‐reducing hydrogenase (Pierce et al. 2008). Both hydrogenase gene clusters were differentially expressed. The transcript levels of genes encoding the electron‐bifurcating hydrogenase genes were reduced (log2FC of −6.0 to −6.6) whereas those of the NADP^+^‐reducing hydrogenase genes were increased (log2FC of +7.9 to +14.3). This is consistent with the finding that the activity of the NADP^+^‐reducing hydrogenase was 15‐fold higher in cells grown on H_2_ + CO_2_, whereas the activity of the electron‐bifurcating hydrogenase was fivefold reduced in comparison to glucose‐grown cells (Table 1). Expression of the Nfn transhydrogenase was increased by a log2FC of +1.7 to 1.9. Genes encoding the EtfABCX complex, potentially involved in electron transfer to quinones, were not differentially expressed when growing on H_2_ + CO_2_.

**TABLE 1:** Enzyme activities of cell‐free extracts prepared from H_2_ + CO_2_–, CO– or glucose‐grown cells.

Enzyme	Reaction catalysed	Activity (U/mg)^a^ in cells grown on		
		H_2_ + CO_2_	CO	Glucose
NADP^+^‐reducing hydrogenase	H_2_ + NADP^+^ → NADPH	1.06 ± 0.17	0.19 ± 0.01	0.07 ± 0.03
Electron‐bifurcating hydrogenase	H_2_ + NAD^+^ + Fd → NADH + Fd^2−^	0.04 ± 0.01	0.09 ± 0.01	0.20 ± 0.02
CODH	CO + Fd → Fd^2−^	2.25 ± 0.25	0.18 ± 0.01	0.39 ± 0.06
NADP^+^‐dependent formate dehydrogenase	Formate + NADP^+^ → NADPH	1.58 ± 0.10	0.77 ± 0.18	0.32 ± 0.03

*Note:* All values are mean ± SD; *n* = 3.

^a^ Enzyme activities were determined as described in 4.4 Enzyme Activity Assays.

*M. thermoacetica* has three Fdhs encoded in its genome, one Fdh is clustered with *ech* genes (putative Fdh–Ech complex) and the other two are annotated as NADPH‐dependent Fdh and cytochrome *b*‐dependent Fdh, respectively. Interestingly, the Fdh–Ech gene cluster also encodes a putative formate transporter. Whereas the *ech* genes were not differentially expressed, transcript levels of genes encoding the Fdh and the putative formate transporter were increased by a log2FC of +2.4 to +3.8 in cells grown on H_2_ + CO_2_. Genes encoding the NADPH‐dependent Fdh were not differentially expressed, but the transcript levels of genes encoding a putative cytochrome *b*‐dependent Fdh were increased by a log2FC of +1.9 to +2.0 (Table S1). The normalised transcript counts of the genes encoding the Fdh subunits of the Fdh–Ech were on average 98 for *fdhA* and 4 for *fdhB*, but only six were found for *fdhA* and none for *fdhB* during growth on glucose. In contrast, the NADPH‐dependent Fdh was expressed to much higher levels with counts of 6628, 3308 and 17,256 for *sfrB2*, *sfrA2* and *fdhF3*, respectively, in cells grown on H_2_ + CO_2_. Approximately two times more transcript counts were detected for *sfrB2* (10,262), *sfrA2* (6277) and *fdhF3* (37,365) when cells grew on glucose. Apparently, the measured NADPH‐dependent Fdh was fivefold higher in H_2_ + CO_2_‐grown cells which shows that reduced transcript counts do not always go along with a reduced enzymatic activity (Table 1). Transcript counts for genes encoding the cytochrome *b*‐dependent Fdh *psrA* (1082), *dmsB4* (240) and *fdoI* (430) were approximately four times higher in H_2_ + CO_2_‐ than in glucose‐grown cells.

Chemiosmotic energy conservation requires an ATP synthase and respiratory enzymes. *M. thermoacetica* has a H^+^–F_1_F_O_–ATP synthase made of eight subunits and ATP synthase transcript levels were increased by a log2FC of +0.8 to +2.1. In addition to the Ech complex, the genome of *M. thermoacetica* encodes a potential membrane‐bound enzyme complex with similarity to NADH dehydrogenase that potentially reduces quinones; transcript levels of the encoding genes were increased in H_2_ + CO_2_‐grown cells, genes encoding the quinone biosynthesis were not differentially expressed during growth on H_2_ + CO_2_.

### 2.3 DEGs Related to the WLP, Energy Conservation and Redox Cycling in CO‐Grown Cells

When grown on CO, the genes encoding the WLP were not differentially expressed compared to glucose‐grown cells. Surprisingly, transcript levels of the monofunctional CODH gene were reduced by a log2FC of −4.1, as seen before with H_2_ + CO_2_ (Table S4). The transcript levels of the CODH/ACS in CO‐grown cells remained similar in comparison to glucose‐grown cells; this is reflected in the reduced CO‐dependent ferredoxin reduction activity as well (Table 1).

Expression of the electron‐bifurcating hydrogenase (log2FC of −6.0 to −6.4) as well as the enzyme activity was reduced in a similar magnitude as seen before with H_2_ + CO_2_ (Table 1; Figure S2). The transcript levels of genes encoding the NADP^+^‐reducing hydrogenase were increased (log2FC of −0.6 to +3.4); the elevated gene expression goes along with a twofold increased enzyme activity of the NADP^+^‐reducing hydrogenase (Table 1).

The genes encoding the cytochrome *b*‐dependent Fdh, EtfABCX and the Nfn complex were not differentially expressed. The genes encoding the ATP synthase were not differentially expressed during growth on CO. The NADH dehydrogenase encoding genes were upregulated by a log2FC of up to +2.4 and, as seen before, the *ech* genes were not differentially expressed but the transcript level for the putative formate transporter and formate dehydrogenase genes were increased by a log2FC of +3.1 to +4.4.

### 2.4 Bioinformatic Analyses of Formate Dehydrogenases of *M. thermoacetica*

Since the formate dehydrogenase is the key enzyme in the WLP, it was purified already in 1983 from *M. thermoacetica* (Yamamoto et al. 1983). The isolated enzyme is a tetramer of two subunits, an α subunit with 96 kDa and a β subunit of 76 kDa. The β subunit is encoded by *sfrB2* and the α subunit is encoded by two genes, *sfrA2* and *fdhF3*. A homodimeric complex of SfrA2 and FdhF3 has a predicted molecular mass of 96 kDa which fits the mass of the isolated α subunit of the NADPH‐dependent Fdh (Yamamoto et al. 1983).

The putative cytochrome *b*‐dependent formate dehydrogenase has not been isolated or characterised. The enzyme is predicted to have three subunits, encoded by the genes *psrA* (Mothe_c03860), *dmsB4* (Mothe_c03870) and *fdoI* (Mothe_c03880) (Pierce et al. 2008). The gene *psrA* (2169 bp) is separated by five nucleotides from the next gene *dmsB4* (696 bp) and *dmsB4* is followed by the gene *fdoI*. The gene *fdoI* overlaps by eight nucleotides with *dmsB4* (681 bp). The genes of the cytochrome *b*‐dependent Fdh are flanked upstream by a gene encoding a putative inner membrane protein and downstream by a gene encoding a glucosyl‐3‐phosphoglycerate synthase. PrsA is a predicted formate dehydrogenase with a molecular mass of 77.6 kDa and has a predicted molybdopterin‐ and a TAT signal sequence. Furthermore, the protein harbours one [4Fe–4S] cluster. DmsB4 is membrane‐bound with a molecular mass of 24.9 kDa and harbours 3 [4Fe–4S] clusters. FdoI has a molecular mass of 25.2 kDa and is suggested to harbour a cytochrome *b* and to contain four transmembrane helices. The physiological role of this Fdh has not been studied.

### 2.5 Bioinformatic Analysis of Enzymes Potentially Involved in Energy Conservation

**FIGURE 3:**
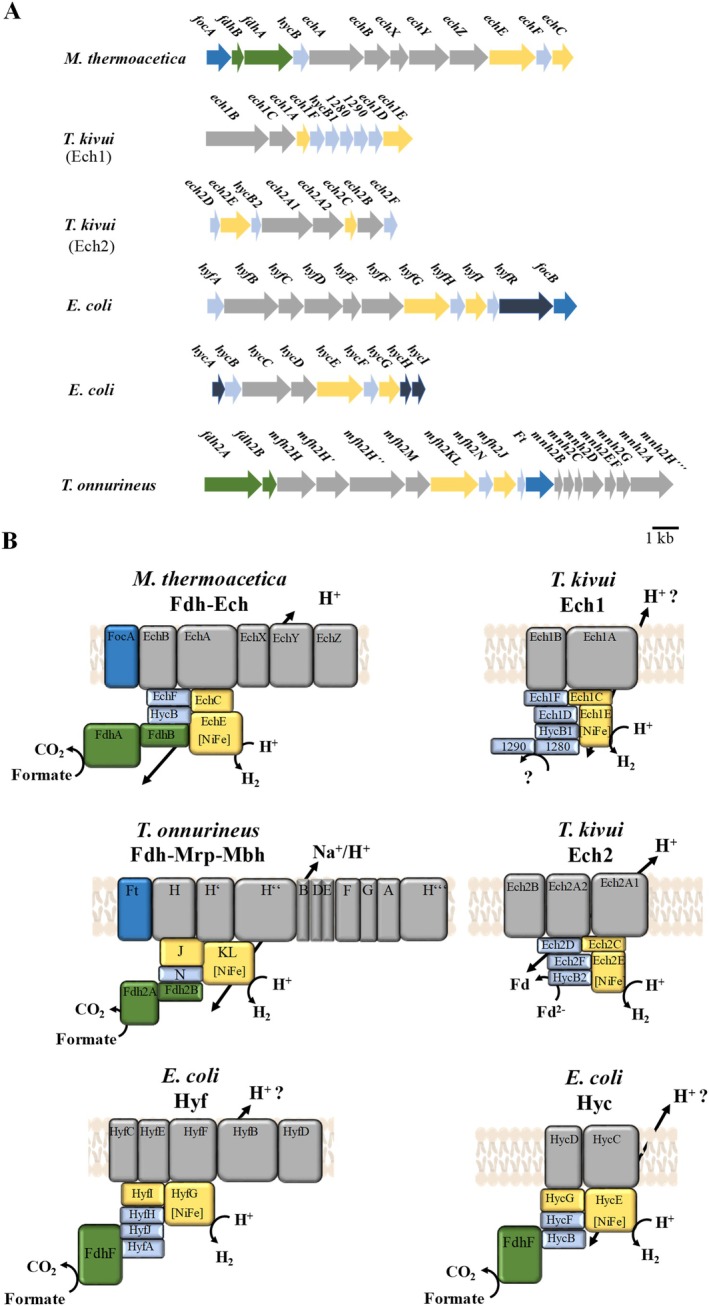
Genetic organisation and models of the molecular architecture of different Ech hydrogenases and formate hydrogenlyases. (A) Genetic organisation of the putative Fdh–Ech from *Moorella thermoacetica*, Ech1 and Ech2 from *Thermoanaerobacter kivui*, Fdh‐Mrp‐Mbh from *T. onnurineus* and the formate hydrogenlyases Hyf and Hyc from *Escherichia* *coli* . Please note, the Fdh of the formate hydrogenlyase complex Hyc or Hyf is not encoded in the same cluster. (B) Models of the molecular architecture of the putative Fdh–Ech from *M. thermoacetica* , Fdh‐Mrp‐Mbh from *Thermoanaerobacter onnurineus*, Ech1 and Ech2 from *T. kivui* and formate hydrogenlyases Hyf and Hyc from *E. coli* . yellow, NiFe hydrogenase subunits; light blue, soluble subunits; grey, membrane‐bound subunits; dark blue, putative formate transporter; green, Fdh subunits; purple, proteins that are not present in the enzyme.

In the transcriptomes, we observed an increased transcript count for possible respiratory enzymes. This prompted us to further investigate the two candidates, the potential NADH dehydrogenase and the Ech complex (Pierce et al. 2008). The unique feature of the *ech* genes is their close proximity to the genes encoding the formate dehydrogenase and a putative formate transporter (Figure 3A). The first gene in the cluster is *focA* (906 bp) encoding the putative formate transporter, followed by *fdhB* (417 bp) and *fdhA*. The gene *fdhA* (1797 bp) overlaps by eight nucleotides with *hycB* (570 bp). Next is the gene *echA* (2022 bp), which overlaps by one nucleotide with *hycB*. *echA* and the following gene *echB* are separated by 12 nucleotides. *echB* (942 bp) is followed by *echX* (651 bp), *echX* is separated from *echB* by 19 bp. Nine nucleotides after *echX* follows *echY* (1479 bp), which is separated by five base pairs from *echZ*. *echZ* (1443 bp) is followed by *echE*. The gene *echE* (1725 bp) is separated by 38 nucleotides from the following gene *echF* (555 bp). *echF* is separated from *echC* (759 bp) by one nucleotide. The genetic organisation implies that Fdh and Ech form a functional complex. The Fdh–Ech gene cluster is flanked upstream by a transcriptional regulator and downstream by genes encoding [NiFe]‐hydrogenase maturation proteins. On the protein level, the deduced subunits of the Fdh–Ech complex have high similarity to the formate hydrogenlyases Hyc and Hyf from *Escherichia* *coli* , Ech1 and Ech2 from *T. kivui* and Fdh‐Mrp‐Mbh from *T. onnurineus* (Table S5). The formate dehydrogenase of the potential Fdh–Ech complex from *M. thermoacetica* consists of two subunits: FdhA, that catalyses formate oxidation and FdhB, that is most likely involved in electron transport towards the [NiFe]‐hydrogenase via HycB. The catalytic subunit FdhA from *M. thermoacetica* shares 42% similarity with the catalytic subunit FdhF from the Hyf or Hyc formate hydrogenlyase of *E. coli* and 47% with the Fdh2A subunit of the Fdh‐Mrp‐Mbh complex. Based on the similarities to the Fdh‐Mrp‐Mbh and Ech complexes it can be hypothesised that the Fdh–Ech complex from *M. thermoacetica* is an ion translocating [NiFe] hydrogenase that uses formate as electron donor for proton reduction to hydrogen gas and that couples electron transport to vectorial proton translocation across the cytoplasmic membrane. Indeed, five genes encode a membrane‐bound, potentially ion translocating module (*echABXYZ*) and four genes code for soluble subunits (*echF*, *hycB*, *echC* and *echE*) (Figure 3A).

**FIGURE 4:**
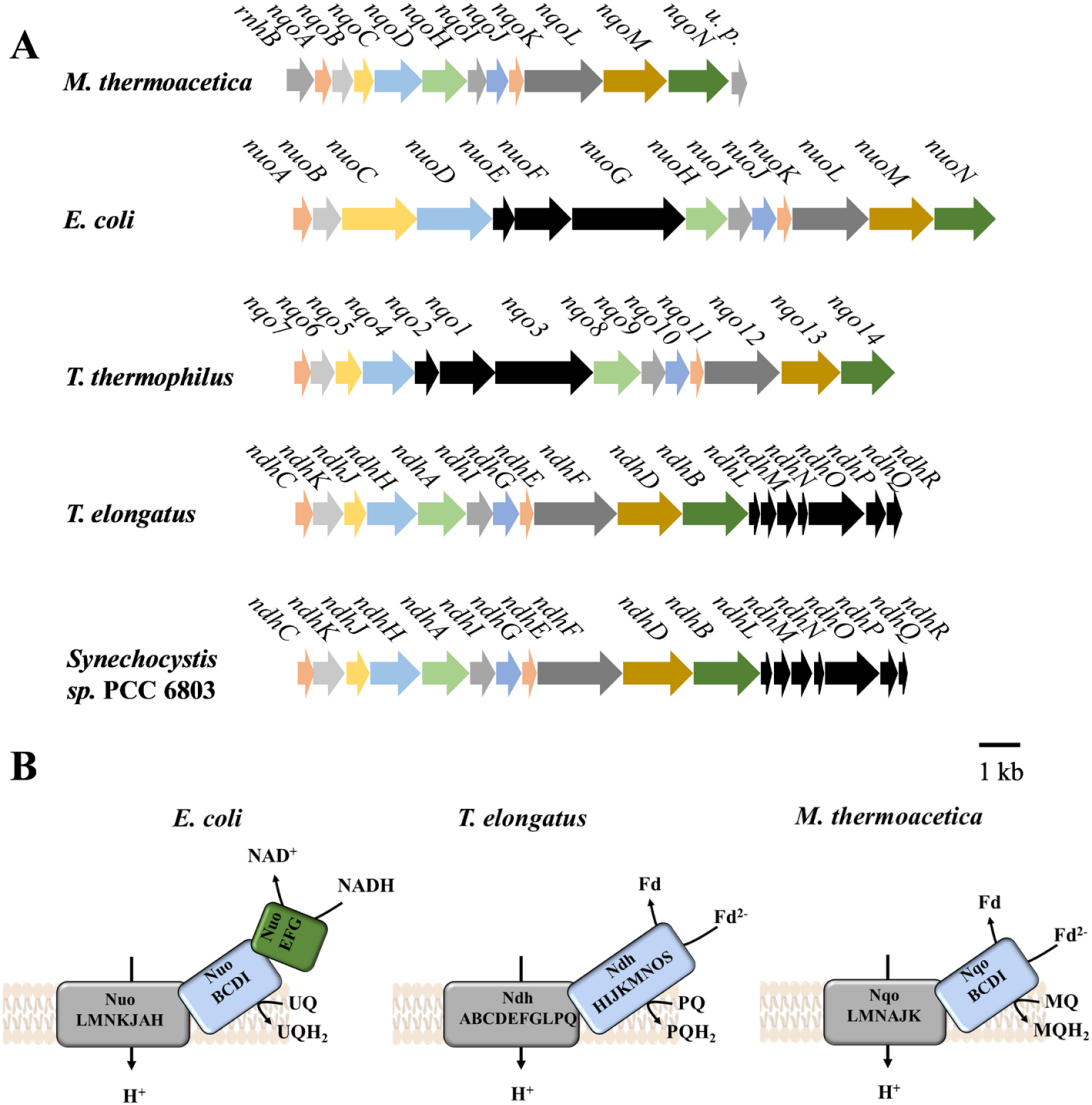
Genetic organisation and models of the architecture of the NADH dehydrogenase or NDH‐1 complex of *Moorella* *thermoacetica*, *Escherichia* *coli* , *Thermoanaerobacter thermophilus*, *Thermoanaerobacter elongatus* and *Synechocystis* sp. (A) Genetic organisation of the NADH dehydrogenase from *M. thermoacetica* , *E. coli* and *T. thermophilus* and NDH‐1 from *T. elongatus* and *Synechocystis* sp. Same colour indicates similar genes, grey colour indicates genes not part of the cluster, black colour indicates missing genes in comparison to the NADH dehydrogenase encoding gene cluster of *M. thermoacetica* . (B) Models of the molecular architecture of the NADH dehydrogenase from *coli* and *M. thermoacetica* and NDH‐1 from *T. elongatus* . light blue, soluble subunits; grey, membrane‐bound subunits; green, NADH oxidising subunits. MQ, menaquinone; MQH_2_, menaquinol; PQ, plastoquinone; PQH_2_, plastoquinol; u. p., unknown protein; UQ, ubiquinone; UQH_2_, ubiquinol.

The second potential respiratory enzyme in *M. thermoacetica* is a predicted complex I‐like NADH‐dehydrogenase (Pierce et al. 2008) (Figure 4A). The encoding gene cluster is flanked upstream by a ribonuclease HII encoding gene and downstream by a gene of unknown function. The first gene of the cluster is *nqoA* (363 bp), which overlaps by 10 nucleotides with the next gene *nqoB*. *nqoB* (501 bp) is separated by one nucleotide from *nqoC* (447 bp). *nqoD* (1116 bp) is three nucleotides apart from *nqoC*. 26 bp separate *nqoD* from the next gene, *nqoH* (1047 bp), which is separated by one nucleotide from *nqoI* (405 bp). The latter overlaps by eight nucleotides with the next gene *nqoJ* (507 bp), which overlaps by four nucleotides with *nqoK* (309 bp). The next gene, *nqoL* (1875 bp), is 31 bp apart from *nqoK*. *nqoL* and *nqoM* (1530 bp) overlap by 4 bp. *nqoM* overlaps by one nucleotide with the last gene of the cluster, *nqoN* (1431 bp).

The potential NADH dehydrogenase from *M. thermoacetica* is very similar to the NADH dehydrogenase from *E. coli* and *Thermus thermophilus* and to the NDH‐1 complex from *Synechocystis* sp. PCC 6803 and *Thermosynechococcus elongatus* (Table S6). However, like the enzymes from *T. elongatus* and *Synechocystis* sp. PCC 6803, the *M. thermoacetica* NADH dehydrogenase lacks the genes *nqoEFG* that encode the NADH oxidising subunits of the complex (Figure 4A). Since it has been shown that the NqoEFG‐lacking (‘headless’) NADH dehydrogenase from *T. elongatus* and/or *Synechocystis* sp. PCC 6803 uses reduced ferredoxin instead of NADH as electron donor (Battchikova et al. 2011; Schuller et al. 2019), we hypothesise that is also true for the enzyme from *M. thermoacetica* . The subunits of the NADH dehydrogenase from *E. coli* are organised into a cytosolic domain including the subunits NuoBCDEFGI and a membrane domain including NuoAHKLMN (Figure 4B). Electron transfer is mostly within the cytosolic domain to a quinone as electron acceptor. NqoDHJK of *M. thermoacetica* are similar to subunits involved in quinone binding. The similarity of the NADH dehydrogenase of *M. thermoacetica* to the Nuo complex of, for example *E. coli* or NDH‐1 of, for example, *T. elongatus* suggests that the NADH dehydrogenase from *M. thermoacetica* is also an ion‐translocating and quinone reducing machine (Figure 4B). Ferredoxin might be the electron donor, but the electron pathway is not as obvious as it is for NDH‐1 from *T. elongatus* . In *T. elongatus* , the subunits NdhS and NdhO are potentially binding ferredoxin as suggested by Laughlin et al. (2019), but these subunits are not encoded in *M. thermoacetica* . Another enzyme complex, the F_420_H_2_ dehydrogenase from *Methanosarcina mazei* , is closely related to NADH dehydrogenases and lacks the NADH oxidising subunits as well (Bäumer et al. 2000; Welte and Deppenmeier 2011). The F_420_H_2_ dehydrogenase from *M. mazei* is a proton translocating, coenzyme F_420_ oxidising enzyme complex (Bäumer et al. 1998).

## 3 Discussion

*M. thermoacetica* does not have a Rnf complex. Based on genomic insights and transcriptome analyses we suggest two novel respiratory enzymes in *M. thermoacetica* , the Fd‐dependent NADH dehydrogenase and a modified version of the Ech complex that does not use ferredoxin but formate as electron donor. A Fdh–Ech‐like complex was previously characterised in the archaeon *T. onnurineus* and demonstrated to mediate H^+^ translocation (Lim et al. 2014). Genes for a similar complex were found in the genomes of over 100 other microorganisms, distributed in several different phyla such as Gammaproteobacteria ( *E. coli* ), Alphaproteobacteria ( *Rhodospirillum rubrum* ) or Firmicutes (*Thermoacetogenium phaeum*) (Keller et al. 2019; Schoelmerich and Müller 2020).

**FIGURE 5:**
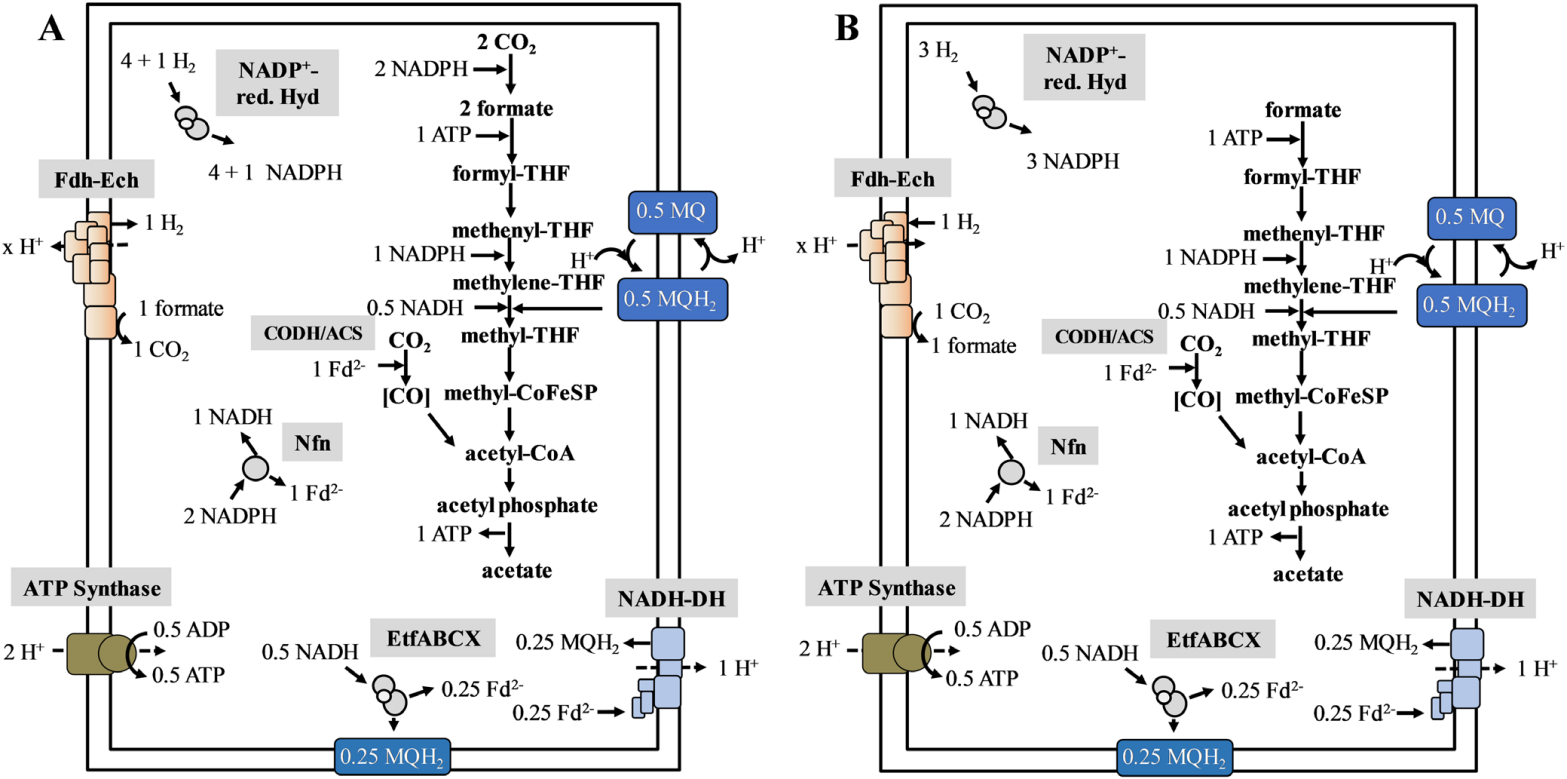
Acetogenesis from H_2_ + CO_2_ in *Moorella thermoacetica*. Acetogenesis from H_2_ + CO_2_ in *M. thermoacetica* at high (A) or low (B) H_2_ partial pressure. For explanations, see text. For the redox loop, see also Figure 6. CODH/ACS, CO dehydrogenase/acetyl‐CoA synthase; Fd^2−^, reduced ferredoxin; NADP^+^‐red; Fdh–Ech, energy‐converting hydrogenase formate dehydrogenase complex; Hyd, NADP^+^‐reducing hydrogenase; MQH_2_, menaquinol; NADH‐DH, NADH dehydrogenase; Nfn, NADH‐dependent ferredoxin:NADP oxidoreductase; THF, tetrahydrofolate; *x*H^+^, number of protons translocated unknown.

The ferredoxin‐dependent NADH dehydrogenase lacks the NADH binding subunits and is suggested to use Fd as electron donor, like the enzymes from *T. elongatus* and *Synechocystis* sp. PCC 6803 (Battchikova et al. 2011; Schuller et al. 2019). The enzymes usually have quinones as electron acceptors and, thus, we postulate that this is the first menaquinone (MQ)‐dependent respiratory enzyme in acetogens. Besides members of the genus *Moorella*, the acetogen *Acetonema longum* also probably has such a Fd‐dependent NADH dehydrogenase. The NADH dehydrogenase may be the third respiratory enzyme in acetogenic bacteria. It appears that not only acetogenic bacteria use such an Fd‐dependent NADH dehydrogenase, but it is also present in sulphate‐reducing bacteria (Pereira et al. 2011). With the data provided we suggest the following models for the biochemistry and the bioenergetics of acetogenesis from H_2_ + CO_2_ or CO in *M. thermoacetica* (Figures 5 and 7).

**FIGURE 6:**
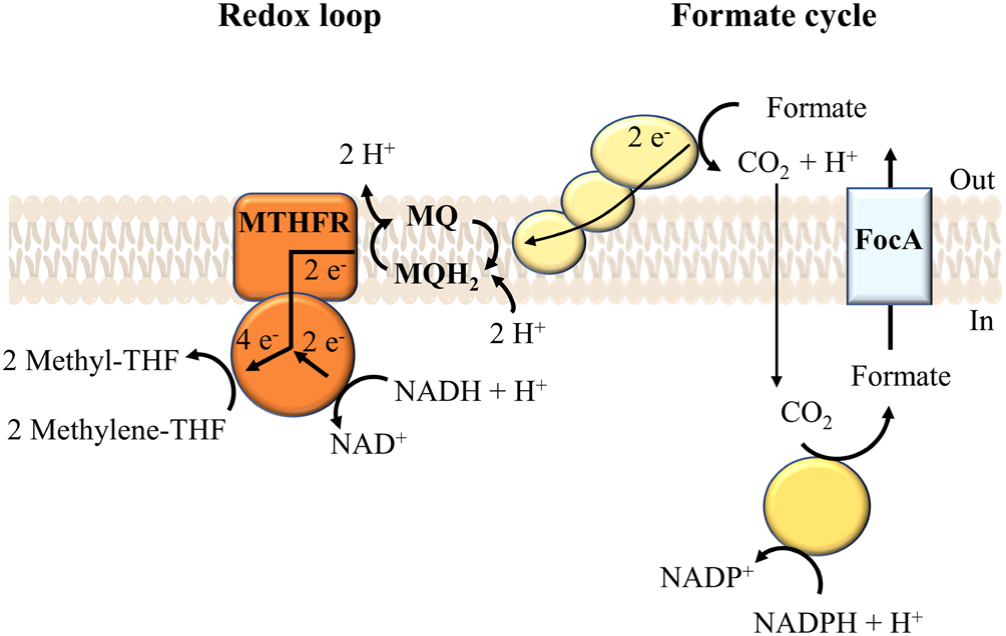
Formate cycle and menaquinone‐dependent redox loop. It is suggested that intracellularly produced formate is actively transported by FocA and oxidised extracellularly by the cytochrome *b*‐dependent Fdh. The resulting CO_2_ is reduced again in the cytoplasm to formate, thereby closing the formate cycle. Electrons derived from formate oxidation are transferred via menaquinone to the MTHFR, establishing a redox loop. The MTHFR uses both reduced menaquinone and NADH in a confurcating reaction to reduce methylene‐THF.

The first step in the methyl branch is the reduction of CO_2_ to formate which poses a thermodynamic hurdle to the cell, as mentioned above (Thauer et al. 1977). NADPH has the same redox potential as NADH under standard conditions but it is kept in more reduced state than NADH in the cell due the fact that is used as major reductant for biosynthetic reactions (Thauer et al. 1977; Huang et al. 2012). The cellular redox potential E' has been determined only for a few bacterial species and is mostly given as −370 mV, in mitochondria it has been determined to be −400 mV (Thauer et al. 1977; Houtkooper et al. 2010). Thus, the redox potential of the NADPH/NADP^+^ couple is at least in the same magnitude as the CO_2_/formate couple. However, the physiological redox potentials of NADPH/NADP^+^ and CO_2_/formate are not known in any acetogen which makes predictions difficult. Anyway, the formate dehydrogenase that was purified from *M. thermoacetica* was cytosolic and used NADPH as (only) reductant (Yamamoto et al. 1983). The enzyme is encoded by Mothe_c23870‐23890 and the corresponding genes were not differentially regulated. At high environmental hydrogen concentrations NADP^+^ is kept in a reduced state by the NADP^+^‐reducing hydrogenase (Rosenbaum and Müller 2024), which is supported by the transcriptomic data and enzyme measurements (Figure 5A). At high hydrogen concentrations, the internal formate concentration is also expected to be elevated. In our model two NADPH are used to drive the reduction of two CO_2_ to two formate. One formate is oxidised to H_2_ + CO_2_ by the putative Fdh–Ech complex which is in line with the transcriptomic data. This reaction kills two birds with one stone: not only is formate taken out of the equilibrium but exergonic electron transfer from formate to protons may lead to vectorial proton transport and the build‐up of an electrochemical proton potential that drives ATP synthesis. The H_2_ produced by the putative Fdh–Ech complex is recaptured by the NADP^+^‐reducing hydrogenase giving one more NADPH. The released CO_2_ is reduced by the CODH/ACS with reduced Fd as reductant (Figure 5A). The second formate is further reduced to methylene‐THF, which requires another NADPH. The remaining two NADPH are oxidised by the Nfn transhydrogenase to reduce one NAD^+^ and Fd. Fd is used to reduce CO_2_ to CO and 0.5 NADH is used to reduce methylene‐THF. The methylene‐THF reductase reaction is still not fully understood. The MTHFR of *M. thermoacetica* is a member of the Type IV class of MTHFR enzymes that has the most complex subunit composition of all MTHFRs (Mock et al. 2014; Öppinger et al. 2022). Type IV enzymes have in addition to the actual MetF subunit MvhD and Hdr subunits and in particular, the presence of HdrA suggests an electron bifurcating mechanism (Kaster et al. 2011). Although NADH oxidation could be demonstrated, electron transfer from NADH to methylene‐THF was not observed which was attributed to the fact that the second electron acceptor was missing. This second electron acceptor was not ferredoxin and it was speculated that a membrane component like a quinone may be the electron acceptor (Mock et al. 2014). We suggest a different mechanism: the MTHFR uses reduced menaquinone (MQ) as a coreductant for methyl‐THF reduction (Figure 5A). Electron transfer from MQH_2_ to methylene‐THF is endergonic (Δ*G*_0_′ = 24.3 kJ/mol; *E*_0_′ = −74 mV) and driven by exergonic electron transfer from NADH to methyl‐THF in an electron confurcating Type IV MTHFR. Oxidation of 0.5 MQH_2_ leads to translocation of one H^+^ to the periplasm and uptake of one H^+^ from the cytoplasm (Figure 6). A similar redox loop was postulated previously in *T. phaeum* (Keller et al. 2019). Depending on the carbon and energy source, a formate‐dependent redox loop is used to reduce methylene‐THF to methyl‐THF, involving proton translocation and *vice versa* (Keller et al. 2019).

Reduced MQ is provided by a chain of redox reactions involving Nfn, EtfABCX and the NADH dehydrogenase (Figure 5A), all required for electron carrier balancing. Altogether, this would lead to at least one H^+^ translocated by the NADH dehydrogenase and one H^+^ by the redox loop, giving 0.5 ATP assuming a H^+^/ATP stoichiometry of 4 (Figure 5A). In addition, protons may be translocated by the Fdh–Ech complex. The reaction is close to equilibrium at standard conditions (Δ*G*_0_′ = −3 kJ/mol). A 10‐fold difference in the educts and products would increase the Δ*G*_0_′ value to 8.7 kJ/mol, a value high enough to allow proton translocation, as demonstrated for *T. onnurineus* (Lim et al. 2014). However, since the number of protons translocated cannot be calculated without knowing the concentration and the magnitude of the membrane potential, we can give no explicit number.

What could be the role of the putative formate transporter and the putative periplasmic cytochrome *b*‐dependent Fdh? They could be part of an energy‐conserving formate cycle. Formate is transported out of the cell by FocA and oxidised by the periplasmic, cytochrome *b*‐dependent Fdh. Electrons are transported to MQ and from there to the MTHFR. Again, as outlined above, this conserves additional energy by a redox loop (Figure 6). A similar mechanism has been proposed for enoyl‐CoA reduction in syntrophic bacteria (Agne et al. 2021). Our hypothesis is in consensus with the transcriptomic data, where we observed increased transcript levels for genes encoding the formate transporter FocA and the cytochrome *b*‐dependent Fdh (Figure 6).

The picture changes at a low H_2_ partial pressure (Figure 5B). Under these conditions, NADPH‐dependent CO_2_ reduction becomes thermodynamically unfavourable. We propose that the putative Fdh–Ech complex now works in reverse; endergonic electron transfer from hydrogen to CO_2_ is driven by the transmembrane electrochemical proton potential allowing growth at unfavourable environmental H_2_ concentrations. In addition, three mol hydrogen are oxidised by the NADPH‐dependent hydrogenase, 2 mol NADPH are converted by the Nfn complex to NADH and reduced ferredoxin; the latter goes into the reduction of CO_2_ in the carbonyl branch. 0.5 mol of NADH are oxidised by the EtfABCX complex, giving 0.25 mol MQH_2_ and 0.25 mol reduced ferredoxin. The latter is oxidised by the NADH dehydrogenase, yielding another 0.25 mol MQH_2_. Finally, 0.5 mol MQH_2_ and NADH are used to reduce methylene‐THF (Figure 5B). In addition, the formate cycle is not active under these conditions. The amount of protons translocated by the NADH dehydrogenase and the redox loop is the same as under high H_2_, but since the Fdh–Ech coupling site is not only lost but reversed, the ATP yield is substantially lower during growth on low H_2_ concentrations (Figure 5B). This may be the reason for the poor growth of *M. thermoacetica* in lab cultures under low H_2_ pressure.

During acetogenesis from CO in *M. thermoacetica* , three of four CO are oxidised and three Fd are reduced, similar to *T. kivui* (Jain et al. 2021). Astonishingly, the monofunctional CO dehydrogenase is downregulated during growth on CO, which contrasts with the finding that the monofunctional CODH was shown to be essential for growth of *T. kivui* on CO (Jain et al. 2021). The downregulation of the monofunctional CODH could be due to two primary reasons. First, monofunctional CODH serves as a protection for CO‐sensitive enzymes, such as electron‐bifurcating hydrogenase, from harmful CO (Jain et al. 2021; Katsyv et al. 2021). Since CO‐sensitive enzymes are downregulated during growth on CO, this protective mechanism does not need to be maintained to the same extent as, for example, during growth on glucose. Second, CODHs are generally very active enzymes, which is why even small amounts of CODH are sufficient to reduce enough ferredoxin for acetogenesis. If the amount of CODH remains similar to, for example, during growth on glucose or is even increased, this could lead to an excess of reduced ferredoxin in the case of *M. thermoacetica* , which could disrupt the redox carrier balance and potentially inhibits acetogenesis.

**FIGURE 7:**
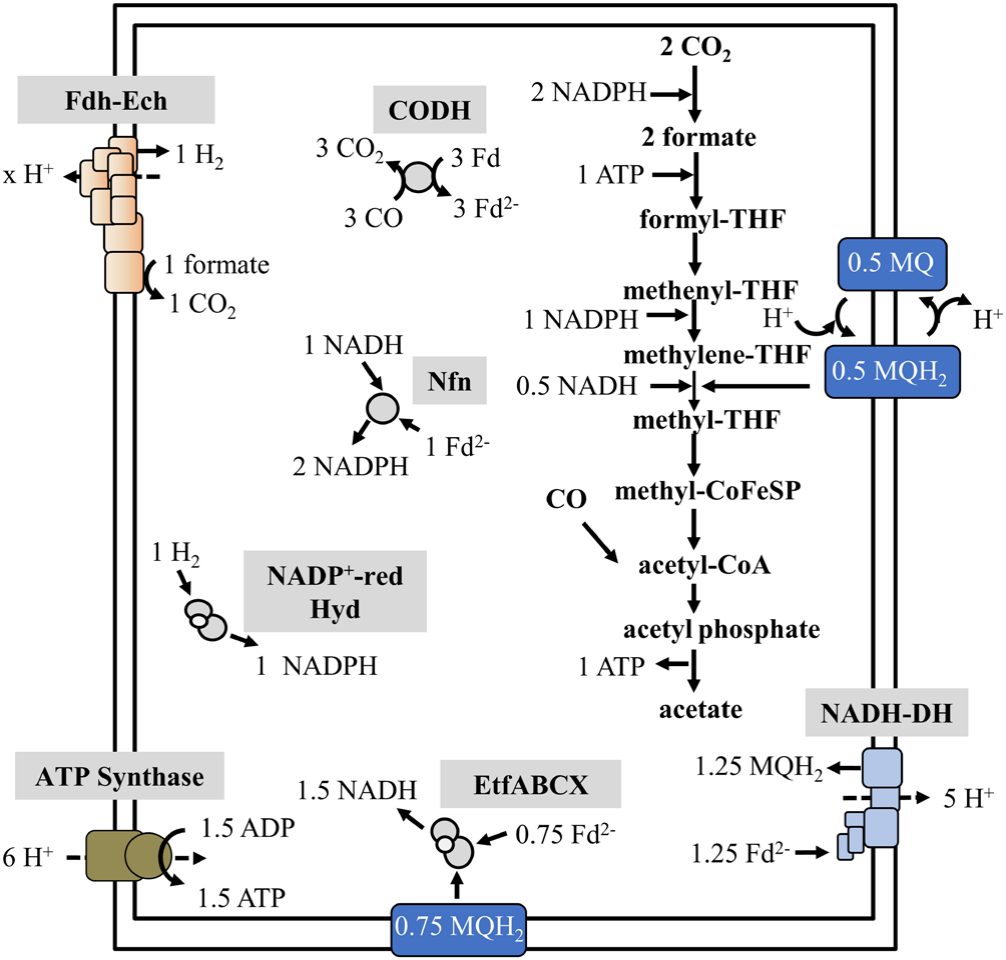
Acetogenesis from CO in *Moorella thermoacetica*. For explanations, see text. CODH/ACS, CO dehydrogenase/acetyl‐CoA synthase; Fd, ferredoxin; Fd^2−^, reduced ferredoxin; Fdh–Ech, energy‐converting hydrogenase formate dehydrogenase complex; MQH_2_, menaquinol; NADH‐DH, NADH dehydrogenase; NADP^+^‐red. Hyd, NADP^+^‐reducing hydrogenase; Nfn, NADH‐dependent ferredoxin:NADP oxidoreductase; THF, tetrahydrofolate; *x*H^+^, number of protons translocated unknown.

The fourth CO is taken up by the CODH/ACS for the formation of acetyl‐CoA (Figure 7). Of the three Fd^2−^ produced, 1.25 Fd^2−^ are oxidised by the NADH dehydrogenase to reduce 1.25 MQ and translocate five protons. Next, 0.75 MQH_2_ and Fd^2−^ are used in an electron‐confurcating reaction to reduce 1.5 NADH by the EtfABCX complex. One NADH and the remaining Fd^2−^ are used by the Nfn to reduce two NADP^+^. In the WLP, two NADPH are used by the formate dehydrogenase to reduce two CO_2_ to two formate. One formate is again oxidised by the putative Fdh–Ech complex releasing H_2_ and CO_2_, coupled to proton translocation. The H_2_ is oxidised by the NADP^+^‐reducing hydrogenase, releasing one more NADPH (Figure 7). The second formate is reduced at the expense of one NADPH to methylene‐THF. For the reduction of methylene‐THF to methyl‐THF catalysed by the MTHFR, 0.5 NADH and 0.5 MQH_2_ are required. During growth on CO, five protons are translocated by the NADH dehydrogenase; some additional protons might be translocated via the putative Fdh–Ech reaction and the formate cycle, giving 1.5 ATP (Figure 7).

## 4 Experimental Procedures

### 4.1 Organism and Cultivation

*M. thermoacetica* DSM 521 was cultivated under anoxic conditions at 55°C in bicarbonate‐buffered complex medium as described previously (Sakimoto et al. 2016). *M. thermoacetica* was grown heterotrophically in 120 mL serum bottles (Glasgerätebau Ochs, Bovenden/Lenglern, Germany) filled with 50 mL complex medium, supplemented with 50 mM glucose under an N_2_ + CO_2_ (80/20 [v/v]) atmosphere, autotrophically on H_2_ + CO_2_ (80/20 [v/v]) or CO (100%) in 120 mL serum bottles (Glasgerätebau Ochs, Bovenden/Lenglern, Germany) filled with 25 mL complex medium with a final pressure of 2 × 10^5^ Pa. The media were prepared using the anaerobic techniques described previously (Hungate 1969; Bryant 1972).

### 4.2 Analytical Methods

Metabolite analyses were carried out using high‐pressure liquid chromatography and gas chromatography as described previously (Schwarz and Müller 2020).

### 4.3 Preparation of Cell‐Free Extract

All steps were carried out in an anaerobic chamber (Coy Laboratories, Grass Lake, USA) containing an N_2_ + H_2_ (95:5 [v/v]) atmosphere. Cultures were harvested at mid‐exponential growth phase by centrifugation (6300*g*, 7 min, 4°C) and washed twice with anoxic harvest buffer (50 mM Tris–HCl (pH 7.5), 20 mM MgSO_4_ × 7 H_2_O, 20% glycerol, 4 mM DTE, 4 μM resazurin). Cell‐free extract was prepared as described previously (Rosenbaum et al. 2021).

### 4.4 Enzyme Activity Assays

All enzyme assays were carried out at 55°C in 1.8 mL anoxic cuvettes (Glasgerätebau Ochs, Bovenden/Lenglern, Germany) filled with enzyme buffer (50 mM Tris/HCl (pH 8), 10 mM NaCl, 2 mM DTE and 4 μM resazurin) with a final volume of 1 mL. One unit is defined as the transfer of 2 μmol electrons min^−1^. All measurements were performed in biological replicates using 50–150 μg of protein. NAD(P)^+^/NAD(P)H was monitored spectrophotometrically at 340 nm (*ε* = 6.3 mM^−1^ cm^−1^) and ferredoxin (Fd) (isolated from *Clostridium pasteurianum* ; Schönheit et al. 1978) at 430 nm (*ε* = 13.1 mM^−1^ cm^−1^) using 2 mM or 30 μM, respectively. Fdh activity was measured under a N_2_ atmosphere (1 × 10^5^ Pa), CODH under a CO atmosphere (1 × 10^5^ Pa) and hydrogenase activity under a H_2_ atmosphere (1 × 10^5^ Pa). The formate‐dependent reactions were started by adding 20 mM formate, H_2_‐dependent reactions were started by adding H_2_ and CO‐dependent reactions were started by adding CO (*n* = 3).

### 4.5 Sampling and Sequencing of RNA

For transcriptome analyses, *M. thermoacetica* was grown on CO, H_2_ + CO_2_ or 50 mM glucose and harvested in the exponential growth phase. The following steps were performed as described (Rosenbaum et al. 2022). Genes with a log2‐fold change of +2/−2 and a *p*‐adjust value of < 0.05 were considered as differentially expressed.

### 4.6 Analysis of Transcriptomic Data and Bioinformatic Analysis

Genes were also manually analysed and curated using the KEGG, KO Database, the Integrated Microbial Genomes–Expert Review (IMG‐ER) database, InterPro, TMHMM and BLAST.

### 4.7 Replicates, Statistical Analysis and Data Representation

All experiments were performed with at least three independent biological replicates. Each biological replicate was initiated with a freshly prepared medium and inoculum. Each biological replicate included three technical duplicates. Data from one representative biological replicate are presented in the figures. Shown values represent means with standard deviation (SD).

## Author Contributions

**Florian P. Rosenbaum:** writing – original draft, investigation, conceptualization, data curation, writing – review and editing, formal analysis. **Anja Poehlein:** data curation, investigation, formal analysis, writing – review and editing. **Rolf Daniel:** data curation, investigation, formal analysis, writing – review and editing. **Volker Müller:** conceptualization, writing – original draft, writing – review and editing.

## Funding

This work was supported by Deutsche Forschungsgemeinschaft by a Reinhart Koselleck project.

## Conflicts of Interest

The authors declare no conflicts of interest.

## Supporting information


**Table S1:** The most upregulated genes of *Moorella thermoacetica* during growth on H_2_ + CO_2_.
**Table S2:** The most downregulated genes of *Moorella thermoacetica* during growth on H_2_ + CO_2_.
**Table S3:** The most upregulated genes of *Moorella thermoacetica* during growth on CO.
**Table S4:** The most downregulated genes of *Moorella thermoacetica* during growth on CO.
**Table S5:** Comparison of the Fdh–Ech from *Moorella thermoacetica* to the Ech1 and Ech2 from *Thermoanaerobacter kivui*, the formate hydrogenlyases Hyf and Hyc from *Escherichia* *coli* and to the Fdh‐Mrp‐Mbh from *Thermoanaerobacter onnurineus*.
**Table S6:** Comparison of the NADH dehydrogenase from *Moorella thermoacetica* , *Escherichia* *coli* , *Thermoanaerobacter thermophilus*, *Synechocystis* sp and *Thermoanaerobacter elongatus*.
**Figure S1:** Overview of transcriptional changes in *Moorella thermoacetica* growing on H_2_ + CO_2_. Depiction of log_2_fold transcriptional changes of genes encoding the WLP, redox balancing enzymes and energy conserving enzymes of cells grown on H_2_ + CO_2_ compared to glucose grown cells (*n* = 3). Electrons are not balanced, oxidation of MQH_2_ by an electron‐bifurcating MTHFR is assumed as well as Fd:quinone oxidoreductase activity of the NADH dehydrogenase. For enzymes containing multiple subunits, the range of expression levels is given.
**Figure S2:** Overview of transcriptional changes in *Moorella thermoacetica* growing on CO. Depiction of log_2_fold transcriptional changes of genes encoding the WLP, redox balancing enzymes and energy conserving enzymes of cells grown on CO versus glucose grown cells (*n* = 3). Electrons are not balanced, oxidation of MQH_2_ by an electron‐bifurcating MTHFR is assumed as well as Fd:quinone oxidoreductase activity of the NADH dehydrogenase. For enzymes containing multiple subunits, the range of expression levels is given.

## Data Availability

Transcriptome data have been deposited in the National Center for Biotechnology Information's (NCBI) Sequence Read Archive (SRA) as BioProject PRJNA1298565 (BioSample no SAMN50279665). All other data of this study are available from the corresponding author upon reasonable request.
