# Supplementary material for: Genomic and Transcriptomic Insights Into the Autotrophic Metabolism on H2 + CO2 or CO of the Thermophilic Acetogenic Model Organism Moorella thermoacetica
Source: Environ Microbiol. 2026 Apr 1;28(4):e70289. doi: 10.1111/1462-2920.70289 (PMC13040438; doi:10.1111/1462-2920.70289)
Supplement: Supplementary file 1 — Table S1: The most upregulated genes of Moorella thermoacetica during growth on H2 + CO2. Table S2: The most downregulated genes of Moorella thermoacetica during growth on H2 + CO2. Table S3: The most upregulated genes of Moorella thermoacetica during growth on CO. Table S4: The most downregulated genes of Moorella thermoacetica during growth on CO. Table S5: Comparison of the Fdh–Ech from Moorella thermoacetica to the Ech1 and Ech2 from Thermoanaerobacter kivui , the formate hydrogenlyases Hyf and Hyc from Escherichia coli and to the Fdh‐Mrp‐Mbh from Thermoanaerobacter onnurineus. Table S6: Comparison of the NADH dehydrogenase from Moorella thermoacetica , Escherichia coli , Thermoanaerobacter thermophilus , Synechocystis sp and Thermoanaerobacter elongatus . Figure S1: Overview of transcriptional changes in Moorella thermoacetica growing on H2 + CO2. Depiction of log2fold transcriptional changes of genes encoding the WLP, redox balancing enzymes and energy conserving enzymes of cells grown on H2 + CO2 compared to glucose grown cells (n = 3). Electrons are not balanced, oxidation of MQH2 by an electron‐bifurcating MTHFR is assumed as well as Fd:quinone oxidoreductase activity of the NADH dehydrogenase. For enzymes containing multiple subunits, the range of expression levels is given. Figure S2: Overview of transcriptional changes in Moorella thermoacetica growing on CO. Depiction of log2fold transcriptional changes of genes encoding the WLP, redox balancing enzymes and energy conserving enzymes of cells grown on CO versus glucose grown cells (n = 3). Electrons are not balanced, oxidation of MQH2 by an electron‐bifurcating MTHFR is assumed as well as Fd:quinone oxidoreductase activity of the NADH dehydrogenase. For enzymes containing multiple subunits, the range of expression levels is given. [file EMI-28-e70289-s001.docx]

**Supplementary Information**

**for the manuscript:**

**Genomic and transcriptomic insights into the autotrophic metabolism on H_2_+CO_2_ or CO of the thermophilic acetogenic model organism *Moorella thermoacetica***

Florian P. Rosenbaum^1^, Anja Poehlein^2^, Rolf Daniel^2^, Volker Müller^1*^

*^1^Department of Molecular Microbiology & Bioenergetics, Institute of Molecular Biosciences, Johann Wolfgang Goethe University, Frankfurt, Germany*

*^2^Genomic and Applied Microbiology & Göttingen Genomics Laboratory, Georg-August University Göttingen, 37077 Göttingen, Germany*

^*^correspondence address: Prof. Volker Müller, Department of Molecular Microbiology & Bioenergetics, Institute of Molecular Biosciences, Johann Wolfgang Goethe University, Frankfurt am Main, Germany; Phone: 49-6979829507; Fax: 49-69-79829306;

E-mail: [vmueller@bio.uni-frankfurt.de](mailto:vmueller@bio.uni-frankfurt.de)

**Supplementary table 1**

**Supplementary table 2**

**Supplementary table 3**

**Supplementary table 4**

**Supplementary table 5**

**Supplementary table 6**

**Supplementary figure 1**

**Supplementary figure 2**

**Supplementary tables**

**Supplementary table 1:** **The most upregulated genes of *M. thermoacetica* during growth on H_2_+CO_2_.**

| **Gene** | **Annotation** | **Substrate** | | **Log_2_ (fold change)** |
| --- | --- | --- | --- | --- |
|  |  | **H_2_+CO_2_^1^** | **Glucose^1^** |  |
| MOTHE_c19190 | NADP^+^-reducing hydrogenase subunit HydD | 3.368 | 0 | 14.3 |
| MOTHE_c19210 | NADP^+^-reducing hydrogenase subunit HndC | 2.752 | 2 | 10.4 |
| MOTHE_c19200 | NADP^+^-reducing hydrogenase subunit HndE | 3.845 | 3 | 10.2 |
| MOTHE_c19180 | NADP^+^-reducing hydrogenase subunit HndA | 14.868 | 14 | 10.0 |
| MOTHE_c19220 | NADP-reducing hydrogenase subunit HndB | 23.071 | 28 | 9.6 |
| MOTHE_c23350 | fructose-1,6-bisphosphatase | 9.019 | 11 | 9.6 |
| MOTHE_c07320 | flagellar biosynthetic protein FliQ | 67 | 0 | 8.1 |
| MOTHE_c19230 | NADP^+^-reducing hydrogenase subunit HndF | 11.808 | 50 | 7.9 |
| MOTHE_c21630 | branched-chain-amino-acid aminotransferase | 5.013 | 92 | 5.7 |
| MOTHE_c07290 | flagellar motor switch protein FliN | 425 | 11 | 5.3 |
| MOTHE_c07330 | flagellar biosynthetic protein FliR | 571 | 16 | 5.2 |
| MOTHE_c06930 | flagellar assembly factor FliW | 311 | 9 | 5.1 |
| MOTHE_c21620 | aminodeoxychorismate synthase component 1 | 12.115 | 366 | 5.0 |
| MOTHE_c07080 | flagellar protein FlaG | 221 | 7 | 4.9 |
| MOTHE_c07430 | flagellar basal-body rod protein FlgG | 608 | 22 | 4.7 |
| MOTHE_c07180 | Yop proteins translocation protein L | 1.439 | 53 | 4.7 |
| MOTHE_c06910 | endo-1,4-beta-xylanase A precursor | 2.296 | 91 | 4.6 |
| MOTHE_c07270 | motility protein B | 632 | 25 | 4.6 |
| MOTHE_c07280 | flagellar FliL protein | 307 | 12 | 4.6 |
| MOTHE_c07170 | flagellar motor switch protein FliG | 1.986 | 80 | 4.6 |
| MOTHE_c07090 | flagellar hook-associated protein 2 | 2.998 | 131 | 4.5 |
| MOTHE_c07160 | flagellar M-ring protein | 3.289 | 145 | 4.5 |
| MOTHE_c15870 | oxalate:formate antiporter | 270 | 11 | 4.5 |
| MOTHE_c07100 | flagellar protein FliS | 237 | 10 | 4.5 |
| MOTHE_c07040 | flagellin C | 16.776 | 765 | 4.4 |
| MOTHE_c07150 | flagellar hook-basal body complex protein FliE | 162 | 7 | 4.3 |
| MOTHE_c00150 | phosphoenolpyruvate-protein phosphotransferase | 10.359 | 453 | 4.3 |
| MOTHE_c15860 | UDP-glucose 4-epimerase | 476 | 24 | 4.3 |
| MOTHE_c15890 | pyruvate synthase subunit PorB | 64.004 | 3.245 | 4.3 |
| MOTHE_c07140 | flagellar basal-body rod protein FlgC | 806 | 43 | 4.2 |
| MOTHE_c04690 | primary amine oxidase precursor | 959 | 50 | 4.2 |
| MOTHE_c08240 | bifunctional protein PyrR | 4.211 | 225 | 4.2 |
| MOTHE_c08250 | aspartate carbamoyltransferase | 6.414 | 336 | 4.1 |
| MOTHE_c07390 | RNA polymerase sigma-D factor | 457 | 26 | 4.1 |
| MOTHE_c00140 | phosphocarrier protein HPr | 293 | 14 | 4.1 |
| MOTHE_c07220 | basal-body rod modification protein FlgD | 303 | 17 | 4.1 |
| MOTHE_c15910 | pyruvate synthase subunit PorD | 50.014 | 2.948 | 4.1 |
| MOTHE_c07240 | flagellar basal-body rod protein FlgG | 1.393 | 84 | 4.0 |
| MOTHE_c08260 | dihydroorotase | 9.386 | 528 | 4.0 |
| MOTHE_c07340 | flagellar biosynthetic protein FlhB | 819 | 49 | 4.0 |
| MOTHE_c15880 | oxalate:formate antiporter | 2.275 | 139 | 4.0 |
| MOTHE_c15900 | NADH-dependent phenylglyoxylate dehydrogenase subunit alpha | 80.272 | 4.887 | 4.0 |
| MOTHE_c06960 | putative pyridoxal phosphate-dependent aminotransferase EpsN | 739 | 46 | 4.0 |
| MOTHE_c06890 | flagellar hook-associated protein 3 | 610 | 38 | 4.0 |
| MOTHE_c07420 | flagellar basal-body rod protein FlgG | 699 | 45 | 3.9 |
| MOTHE_c07200 | flagellar FliJ protein | 370 | 24 | 3.9 |
| MOTHE_c07300 | flagellar biosynthesis protein FliO | 288 | 19 | 3.9 |
| MOTHE_c22520 | formate dehydrogenase H | 98 | 6 | 3.8 |
| MOTHE_c17520 | motility protein B | 318 | 23 | 3.8 |
| MOTHE_c07410 | YceG-like family protein | 368 | 26 | 3.7 |
| MOTHE_c07260 | chemotaxis protein PomA | 999 | 74 | 3.7 |
| MOTHE_c07210 | flagellar hook-length control protein FliK | 4.340 | 325 | 3.7 |
| MOTHE_c00130 | PTS system fructose-specific EIIABC component | 1.879 | 119 | 3.7 |
| MOTHE_c07190 | putative ATP synthase YscN | 4.109 | 314 | 3.7 |
| MOTHE_c07310 | flagellar biosynthetic protein FliP precursor | 1.330 | 101 | 3.7 |
| MOTHE_c00120 | PTS system fructose-specific EIIBC component | 8.405 | 549 | 3.7 |
| MOTHE_c17510 | chemotaxis protein PomA | 285 | 22 | 3.6 |
| MOTHE_c07130 | flagellar basal body rod protein FlgB | 536 | 43 | 3.6 |
| MOTHE_c06880 | flagellar hook-associated protein 1 | 1.677 | 135 | 3.6 |
| MOTHE_c07370 | flagellum site-determining protein YlxH | 940 | 78 | 3.6 |
| MOTHE_c07350 | flagellar biosynthesis protein FlhA | 1.770 | 147 | 3.6 |
| MOTHE_c15240 | stage III sporulation protein AE precursor | 82 | 7 | 3.6 |
| MOTHE_c20900 | spore germination protein B1 | 1.182 | 102 | 3.5 |
| MOTHE_c25510 | 50S ribosomal protein L10 | 4.376 | 381 | 3.5 |
| MOTHE_c20930 | phosphoribosylglycinamide formyltransferase | 2.614 | 230 | 3.5 |
| MOTHE_c22540 | putative formate transporter 1 | 2.461 | 220 | 3.5 |
| MOTHE_c06950 | UDP-glucose 4-epimerase | 1.000 | 93 | 3.4 |
| MOTHE_c20950 | amidophosphoribosyltransferase precursor | 7.792 | 739 | 3.4 |
| MOTHE_c20990 | phosphoribosylaminoimidazole-succinocarboxamide synthase | 4.116 | 400 | 3.4 |
| MOTHE_c20890 | spore germination protein YndE | 257 | 27 | 3.2 |
| MOTHE_c25400 | 50S ribosomal protein L4 | 5.467 | 581 | 3.2 |
| MOTHE_c05610 | tagatose-6-phosphate kinase | 552 | 55 | 3.2 |
| MOTHE_c25380 | 50S ribosomal protein L2 | 8.454 | 911 | 3.2 |
| MOTHE_c25410 | 50S ribosomal protein L3 | 9.603 | 1.041 | 3.2 |
| MOTHE_c25440 | elongation factor G | 41.673 | 4.596 | 3.2 |
| MOTHE_c07380 | flagellar brake protein YcgR | 754 | 85 | 3.1 |
| MOTHE_c07250 | flagellar protein (FlbD) | 92 | 10 | 3.1 |
| MOTHE_c07360 | flagellar biosynthesis protein FlhF | 1.414 | 164 | 3.1 |
| MOTHE_c25360 | 50S ribosomal protein L22 | 3.666 | 423 | 3.1 |
| MOTHE_c25390 | 50S ribosomal protein L23 | 1.863 | 219 | 3.1 |
| MOTHE_c25350 | 30S ribosomal protein S3 | 6.423 | 754 | 3.1 |
| MOTHE_c25310 | 50S ribosomal protein L14 | 1.236 | 146 | 3.1 |
| MOTHE_c25370 | 30S ribosomal protein S19 | 1.008 | 122 | 3.0 |
| MOTHE_c25500 | 50S ribosomal protein L7/L12 | 3.082 | 380 | 3.0 |
| MOTHE_c05160 | citrate transporter | 4.454 | 556 | 3.0 |
| MOTHE_c20960 | phosphoribosylformylglycinamidine synthase 2 | 13.145 | 1.650 | 3.0 |
| MOTHE_c13320 | universal stress protein family protein | 272 | 33 | 3.0 |
| MOTHE_c25320 | 30S ribosomal protein S17 | 552 | 67 | 3.0 |
| MOTHE_c25290 | 50S ribosomal protein L5 | 3.785 | 477 | 3.0 |
| MOTHE_c10870 | putative endonuclease 4 | 170 | 21 | 3.0 |
| MOTHE_c09270 | tRNA (guanine-N(1)-)-methyltransferase | 16.834 | 2.169 | 3.0 |
| MOTHE_c25430 | elongation factor Tu-B | 35.241 | 4.707 | 2.9 |
| MOTHE_c08290 | orotidine 5'-phosphate decarboxylase | 2.276 | 278 | 2.9 |
| MOTHE_c13340 | Trk system potassium uptake protein TrkA | 490 | 67 | 2.9 |
| MOTHE_c25420 | 30S ribosomal protein S10 | 2.297 | 316 | 2.9 |
| MOTHE_c04960 | putative zinc metalloprotease Rip3 | 573 | 78 | 2.8 |
| MOTHE_c05150 | leucine--tRNA ligase | 19.314 | 2.682 | 2.8 |
| MOTHE_c22740 | putative permease | 187 | 24 | 2.8 |
| MOTHE_c12760 | glutamate synthase [NADPH] large chain | 1.427 | 199 | 2.8 |
| MOTHE_c06870 | FlgN protein | 452 | 62 | 2.8 |
| MOTHE_c25300 | 50S ribosomal protein L24 | 1.407 | 197 | 2.8 |
| MOTHE_c10040 | DNA polymerase III PolC-type | 11.996 | 1.697 | 2.8 |
| MOTHE_c25340 | 50S ribosomal protein L16 | 2.993 | 423 | 2.8 |
| MOTHE_c09570 | enterobactin exporter EntS | 307 | 42 | 2.8 |
| MOTHE_c09180 | ABC-2 family transporter protein | 4.508 | 641 | 2.8 |
| MOTHE_c23970 | macrolide export protein MacA | 964 | 137 | 2.8 |
| MOTHE_c13440 | putative spore protein YtfJ | 124 | 17 | 2.8 |
| MOTHE_c20980 | phosphoribosylformylglycinamidine synthase subunit PurS | 759 | 111 | 2.8 |
| MOTHE_c09260 | ribosome maturation factor RimM | 14.036 | 2.072 | 2.8 |
| MOTHE_c05130 | putative nicotinate-nucleotide adenylyltransferase | 3.514 | 521 | 2.7 |
| MOTHE_c09170 | putative ABC transporter ATP-binding protein YxlF | 5.525 | 811 | 2.7 |
| MOTHE_c20020 | carbon starvation protein A | 71.214 | 10.622 | 2.7 |
| MOTHE_c25250 | 50S ribosomal protein L18 | 2.112 | 316 | 2.7 |
| MOTHE_c13330 | Trk system potassium uptake protein TrkA | 200 | 29 | 2.7 |
| MOTHE_c21560 | uroporphyrinogen decarboxylase | 132 | 19 | 2.7 |
| MOTHE_c00110 | tagatose-6-phosphate kinase | 2.029 | 232 | 2.7 |
| MOTHE_c25330 | 50S ribosomal protein L29 | 346 | 51 | 2.7 |
| MOTHE_c22070 | putative diguanylate cyclase YeaJ | 1.233 | 185 | 2.7 |
| MOTHE_c22060 | response regulator PleD | 1.078 | 162 | 2.7 |
| MOTHE_c10030 | proline--tRNA ligase | 14.466 | 2.223 | 2.7 |
| MOTHE_c25270 | 30S ribosomal protein S8 | 757 | 112 | 2.7 |
| MOTHE_c15930 | ATP-dependent zinc metalloprotease FtsH | 11.365 | 1.737 | 2.7 |
| MOTHE_c06860 | anti-sigma-28 factor. FlgM | 328 | 50 | 2.7 |
| MOTHE_c09600 | flagellar biosynthetic protein FlhB | 4 | 0 | 2.7 |
| MOTHE_c18420 | PhoU domain protein | 223 | 34 | 2.7 |
| MOTHE_c05620 | D-ribose-binding periplasmic protein precursor | 637 | 95 | 2.7 |
| MOTHE_c13980 | electron transport complex subunit RsxB | 14.842 | 2.009 | 2.6 |
| MOTHE_c09150 | putative ECF RNA polymerase sigma factor SigI | 2.211 | 354 | 2.6 |
| MOTHE_c17290 | multifunctional cyclase-dehydratase-3-O-methyl transferase TcmN | 678 | 108 | 2.6 |
| MOTHE_c20970 | phosphoribosylformylglycinamidine synthase 1 | 4.003 | 654 | 2.6 |
| MOTHE_c00350 | sigmaK-factor processing regulatory protein BofA | 52 | 8 | 2.6 |
| MOTHE_c06970 | phosphoribosylamine--glycine ligase | 172 | 27 | 2.6 |
| MOTHE_c02200 | OPT oligopeptide transporter protein | 28.668 | 4.829 | 2.6 |
| MOTHE_c20940 | phosphoribosylformylglycinamidine cyclo-ligase | 4.708 | 791 | 2.6 |
| MOTHE_c25280 | 30S ribosomal protein S14 type Z | 250 | 40 | 2.5 |
| MOTHE_c12770 | glutamine synthetase | 2.962 | 503 | 2.5 |
| MOTHE_c09240 | 30S ribosomal protein S16 | 2.118 | 370 | 2.5 |
| MOTHE_c05140 | ribosomal silencing factor RsfS | 998 | 173 | 2.5 |
| MOTHE_c12740 | iron-sulfur protein | 695 | 123 | 2.5 |
| MOTHE_c09230 | signal recognition particle protein | 36.802 | 6.610 | 2.5 |
| MOTHE_c13300 | putative amino acid permease YhdG | 1.550 | 278 | 2.5 |
| MOTHE_c09320 | transcriptional regulatory protein DegU | 1.271 | 229 | 2.5 |
| MOTHE_c25240 | 30S ribosomal protein S5 | 3.735 | 672 | 2.5 |
| MOTHE_c10910 | glutamine transport ATP-binding protein GlnQ | 1.882 | 346 | 2.4 |
| MOTHE_c09560 | multidrug resistance protein 3 | 426 | 78 | 2.4 |
| MOTHE_c10010 | regulator of sigma-W protease RasP | 5.269 | 991 | 2.4 |
| MOTHE_c21000 | adenylosuccinate lyase | 8.721 | 1.633 | 2.4 |
| MOTHE_c14010 | dinitrogenase iron-molybdenum cofactor | 3.850 | 603 | 2.4 |
| MOTHE_c10130 | riboflavin biosynthesis protein RibF | 1.518 | 283 | 2.4 |
| MOTHE_c13990 | anaerobic sulfite reductase subunit C | 16.523 | 2.595 | 2.4 |
| MOTHE_c12750 | glutamate synthase [NADPH] large chain precursor | 2.255 | 421 | 2.4 |
| MOTHE_c10670 | EamA-like transporter family protein | 144 | 26 | 2.4 |
| MOTHE_c10640 | threonine-phosphate decarboxylase | 968 | 182 | 2.4 |
| MOTHE_c14020 | dinitrogenase iron-molybdenum cofactor | 5.263 | 838 | 2.4 |
| MOTHE_c22530 | formate dehydrogenase H | 4 | 0 | 2.4 |
| MOTHE_c25260 | 50S ribosomal protein L6 | 3.234 | 614 | 2.4 |
| MOTHE_c12720 | glutamate synthase [NADPH] large chain precursor | 766 | 145 | 2.4 |
| MOTHE_c20880 | spore germination protein B3 precursor | 323 | 60 | 2.4 |
| MOTHE_c04950 | stage II sporulation protein Q | 997 | 192 | 2.4 |
| MOTHE_c23210 | 2,3-dimethylmalate dehydratase large subunit | 1.306 | 251 | 2.4 |
| MOTHE_c21010 | N5-carboxyaminoimidazole ribonucleotide mutase | 3.140 | 608 | 2.4 |
| MOTHE_c16920 | glycine betaine/carnitine/choline-binding protein OpuCC precursor | 1.148 | 216 | 2.3 |
| MOTHE_c12520 | muramidase-2 precursor | 32 | 5 | 2.3 |
| MOTHE_c07500 | flagellar motor switch protein FliN | 1.846 | 362 | 2.3 |
| MOTHE_c02250 | 2,5-dihydroxypyridine 5,6-dioxygenase | 14.393 | 2.839 | 2.3 |
| MOTHE_c20600 | spore coat protein F precursor | 185 | 35 | 2.3 |
| MOTHE_c10630 | L-threonine kinase | 1.291 | 256 | 2.3 |
| MOTHE_c25150 | 30S ribosomal protein S13 | 1.637 | 328 | 2.3 |
| MOTHE_c09430 | NAD(P)H-quinone oxidoreductase chain 4 1 | 7.199 | 1.452 | 2.3 |
| MOTHE_c25450 | 30S ribosomal protein S7 | 8.648 | 1.770 | 2.3 |
| MOTHE_c15000 | NAD kinase | 2.527 | 516 | 2.3 |
| MOTHE_c23190 | 2,3-dimethylmalate dehydratase small subunit | 1.836 | 378 | 2.3 |
| MOTHE_c09310 | signal transduction histidine-protein kinase/phosphatase DegS | 2.756 | 564 | 2.3 |
| MOTHE_c07120 | SpoVT / AbrB like domain protein | 30 | 5 | 2.3 |
| MOTHE_c07490 | flagellar motor switch protein FliM | 1.326 | 279 | 2.2 |
| MOTHE_c12880 | aspartokinase | 8.902 | 1.885 | 2.2 |
| MOTHE_c14990 | arginine repressor | 370 | 77 | 2.2 |
| MOTHE_c23980 | putative multidrug resistance protein EmrK | 442 | 91 | 2.2 |
| MOTHE_c17310 | phosphomethylpyrimidine synthase | 759 | 159 | 2.2 |
| MOTHE_c25190 | methionine aminopeptidase 1 | 5.500 | 1.168 | 2.2 |
| MOTHE_c12870 | N-acetylmuramoyl-L-alanine amidase sle1 precursor | 1.563 | 337 | 2.2 |
| MOTHE_c25170 | translation initiation factor IF-1 | 395 | 83 | 2.2 |
| MOTHE_c20920 | bifunctional purine biosynthesis protein PurH | 9.239 | 1.981 | 2.2 |
| MOTHE_c07780 | glucokinase | 3.337 | 729 | 2.2 |
| MOTHE_c23200 | 2,3-dimethylmalate dehydratase large subunit | 2.054 | 447 | 2.2 |
| MOTHE_c23850 | ferredoxin--NADP reductase | 2.101 | 455 | 2.2 |
| MOTHE_c25490 | DNA-directed RNA polymerase subunit beta | 12.481 | 2.752 | 2.2 |
| MOTHE_c13690 | DMSO reductase anchor subunit (DmsC) | 658 | 141 | 2.2 |
| MOTHE_c09440 | NADH-quinone oxidoreductase subunit N | 7.408 | 1.620 | 2.2 |
| MOTHE_c01480 | aspartate 1-decarboxylase precursor | 268 | 54 | 2.2 |
| MOTHE_c19250 | ferredoxin-1 | 213 | 47 | 2.2 |
| MOTHE_c25460 | 30S ribosomal protein S12 | 8.788 | 1.973 | 2.2 |
| MOTHE_c08670 | ribulose-phosphate 3-epimerase | 727 | 165 | 2.1 |
| MOTHE_c10900 | inner membrane amino-acid ABC transporter permease protein YecS | 1.935 | 440 | 2.1 |
| MOTHE_c00190 | D-3-phosphoglycerate dehydrogenase | 11.865 | 2.735 | 2.1 |
| MOTHE_c10760 | tRNA dimethylallyltransferase | 1.065 | 246 | 2.1 |
| MOTHE_c17320 | phosphomethylpyrimidine synthase | 650 | 145 | 2.1 |
| MOTHE_c23170 | 2-isopropylmalate synthase | 6.649 | 1.537 | 2.1 |
| MOTHE_c15010 | 16S/23S rRNA (cytidine-2'-O)-methyltransferase TlyA | 1.931 | 449 | 2.1 |
| MOTHE_c12240 | putative dimethyl sulfoxide reductase chain YnfF precursor | 5.469 | 1.286 | 2.1 |
| MOTHE_c23990 | multidrug export protein EmrB | 952 | 220 | 2.1 |
| MOTHE_c24600 | ATP synthase subunit delta | 7.662 | 1.820 | 2.1 |
| MOTHE_c00100 | glucitol operon repressor | 2.497 | 498 | 2.1 |
| MOTHE_c25140 | 30S ribosomal protein S11 | 1.660 | 396 | 2.0 |
| MOTHE_c17300 | nicotinate-nucleotide--dimethylbenzimidazole phosphoribosyltransferase | 885 | 209 | 2.0 |
| MOTHE_c08960 | acetate kinase | 9.897 | 2.403 | 2.0 |
| MOTHE_c10410 | ribonuclease Y | 4.811 | 1.152 | 2.0 |
| MOTHE_c11730 | 5-methyltetrahydrofolate:corrinoid/iron-sulfur protein co-methyltransferase | 25.824 | 6.258 | 2.0 |
| MOTHE_c19730 | glycogen synthase | 107 | 25 | 2.0 |
| MOTHE_c23180 | 3-isopropylmalate dehydrogenase | 5.388 | 1.329 | 2.0 |
| MOTHE_c09420 | NADH-quinone oxidoreductase subunit 12 | 7.205 | 1.765 | 2.0 |
| MOTHE_c14770 | methyl-accepting chemotaxis protein McpA | 592 | 144 | 2.0 |
| MOTHE_c10280 | 4-hydroxy-tetrahydrodipicolinate synthase | 1.128 | 278 | 2.0 |
| MOTHE_c09280 | 50S ribosomal protein L19 | 3.596 | 879 | 2.0 |
| MOTHE_c07480 | chemotaxis protein CheY | 365 | 90 | 2.0 |
| MOTHE_c16930 | glycine betaine/carnitine/choline transport system permease protein OpuCD | 587 | 141 | 2.0 |
| MOTHE_c13700 | anaerobic dimethyl sulfoxide reductase chain B | 480 | 117 | 2.0 |
| MOTHE_c03050 | NADH-dependent phenylglyoxylate dehydrogenase subunit gamma | 7 | 1 | 2.0 |
| MOTHE_c10110 | bifunctional oligoribonuclease and PAP phosphatase NrnA | 2.594 | 649 | 2.0 |
| MOTHE_c08660 | putative ribosome biogenesis GTPase RsgA | 1.702 | 425 | 2.0 |
| MOTHE_c12730 | NADH-dependent phenylglyoxylate dehydrogenase subunit epsilon | 2.112 | 526 | 2.0 |
| MOTHE_c24590 | ATP synthase subunit alpha | 28.937 | 7.292 | 2.0 |
| MOTHE_c13130 | isopentenyl-diphosphate delta-isomerase | 1.941 | 490 | 2.0 |
| MOTHE_c13590 | SpoVA protein | 4 | 0 | 2.0 |
| MOTHE_c10020 | 4-hydroxy-3-methylbut-2-en-1-yl diphosphate synthase | 11.776 | 2.992 | 2.0 |
| MOTHE_c10590 | cobalamin biosynthesis protein CbiB | 2.005 | 509 | 2.0 |
| MOTHE_c18510 | HTH-type transcriptional activator CmpR | 512 | 129 | 2.0 |

^1^Mean values of normalized read counts.

**Supplementary table 2: The most downregulated genes of *M. thermoacetica* during growth on H_2_+CO_2_.**

| **Gene** | **Annotation** | **Substrate** | | **Log2 (fold change)** |
| --- | --- | --- | --- | --- |
|  |  | **H_2_+CO_2_^1^** | **Glucose^1^** |  |
| MOTHE_c14240 | ferrous iron transport protein A | 3 | 1.929 | -9.0 |
| MOTHE_c14230 | FeoA domain protein | 7 | 2.641 | -8.3 |
| MOTHE_c14220 | ferrous iron transport protein B | 218 | 52.267 | -7.8 |
| MOTHE_c03560 | KHG/KDPG aldolase | 80 | 15.443 | -7.6 |
| MOTHE_c11590 | FeoA domain protein | 114 | 16.978 | -7.2 |
| MOTHE_c03550 | 2-dehydro-3-deoxygluconokinase | 151 | 21.736 | -7.1 |
| MOTHE_c03540 | K^+^-stimulated pyrophosphate-energized sodium pump | 169 | 23.805 | -7.1 |
| MOTHE_c02130 | spore cortex-lytic enzyme precursor | 0 | 15 | -6.8 |
| MOTHE_c14210 | cyclic di-GMP phosphodiesterase response regulator RpfG | 78 | 8.616 | -6.7 |
| MOTHE_c17280 | Electron bifurcating hydrogenase subunit HydC | 21 | 2.493 | -6.6 |
| MOTHE_c17270 | Electron bifurcating hydrogenase subunit HydB | 193 | 17.444 | -6.3 |
| MOTHE_c11580 | ferrous iron transport protein B | 1.868 | 123.223 | -6.0 |
| MOTHE_c17260 | Electron bifurcating hydrogenase subunit HydA | 230 | 16.489 | -6.0 |
| MOTHE_c04090 | Hsp20/alpha crystallin family protein | 133 | 5.233 | -5.3 |
| MOTHE_c15520 | ultraviolet N-glycosylase/AP lyase | 30 | 1.056 | -5.1 |
| MOTHE_c23830 | putative FAD-linked oxidoreductase | 1.745 | 58.014 | -5.0 |
| MOTHE_c19130 | reverse rubrerythrin-1 | 2.038 | 79.957 | -4.9 |
| MOTHE_c23820 | anaerobic glycerol-3-phosphate dehydrogenase subunit C | 1.416 | 39.923 | -4.8 |
| MOTHE_c23810 | lactate utilization protein C | 415 | 11.556 | -4.8 |
| MOTHE_c21480 | HTH-type transcriptional regulator CymR | 31 | 1.294 | -4.8 |
| MOTHE_c16600 | HTH-type transcriptional regulator CymR | 803 | 22.431 | -4.8 |
| MOTHE_c21980 | transposase | 51 | 1.745 | -4.6 |
| MOTHE_c20120 | O-acetylserine sulfhydrylase | 1.150 | 28.558 | -4.6 |
| MOTHE_c23800 | lactate utilization protein B | 1.552 | 38.069 | -4.6 |
| MOTHE_c16150 | RNA polymerase sigma factor RpoD | 13 | 318 | -4.6 |
| MOTHE_c04100 | calcium-transporting ATPase | 20 | 535 | -4.6 |
| MOTHE_c23780 | glycolate permease GlcA | 820 | 19.355 | -4.5 |
| MOTHE_c22300 | sensor histidine kinase YehU | 217 | 4.682 | -4.4 |
| MOTHE_c15680 | primary amine oxidase precursor | 10 | 228 | -4.4 |
| MOTHE_c11280 | alkaline phosphatase precursor | 8.807 | 203.815 | -4.4 |
| MOTHE_c06370 | glucitol operon repressor | 57 | 1.119 | -4.3 |
| MOTHE_c16590 | cysteine desulfurase IscS | 8.228 | 145.065 | -4.1 |
| MOTHE_c16580 | iron-sulfur cluster assembly scaffold protein IscU | 1.119 | 19.403 | -4.1 |
| MOTHE_c22310 | sensory transduction protein LytR | 97 | 1.637 | -4.1 |
| MOTHE_c20130 | carbon monoxide dehydrogenase 1 | 2.319 | 39.201 | -4.1 |
| MOTHE_c23790 | HTH-type transcriptional regulator LutR | 801 | 13.263 | -4.0 |
| MOTHE_c17130 | cysteine synthase | 920 | 14.251 | -3.9 |
| MOTHE_c08870 | peroxide-responsive repressor PerR | 336 | 5.039 | -3.9 |
| MOTHE_c08860 | high-affinity zinc uptake system binding-protein ZnuA precursor | 970 | 13.775 | -3.8 |
| MOTHE_c12670 | high molecular weight rubredoxin | 57 | 962 | -3.8 |
| MOTHE_c20150 | bicarbonate transport system permease protein CmpB | 320 | 4.529 | -3.8 |
| MOTHE_c20080 | sulfur carrier protein ThiS | 105 | 1.405 | -3.7 |
| MOTHE_c06680 | Gnt-II system L-idonate transporter | 66 | 857 | -3.7 |
| MOTHE_c20140 | putative aliphatic sulfonates-binding protein precursor | 568 | 6.975 | -3.6 |
| MOTHE_c15700 | nucleotidyltransferase domain protein | 5 | 66 | -3.6 |
| MOTHE_c06390 | ribose import ATP-binding protein RbsA | 32 | 410 | -3.6 |
| MOTHE_c21490 | cytochrome bd ubiquinol oxidase subunit 1 | 187 | 3.916 | -3.5 |
| MOTHE_c06670 | 4-hydroxythreonine-4-phosphate dehydrogenase 2 | 144 | 1.651 | -3.5 |
| MOTHE_c23300 | zinc ribbon domain protein | 12 | 147 | -3.5 |
| MOTHE_c08850 | hemin import ATP-binding protein HmuV | 923 | 9.982 | -3.4 |
| MOTHE_c20110 | putative adenylyltransferase/sulfurtransferase MoeZ | 4.858 | 51.540 | -3.4 |
| MOTHE_c02530 | HTH-type transcriptional regulator ImmR | 28 | 291 | -3.4 |
| MOTHE_c06380 | D-allose-binding periplasmic protein precursor | 117 | 1.189 | -3.3 |
| MOTHE_c14290 | ATP-dependent (S)-NAD(P)H-hydrate dehydratase | 1.433 | 15.452 | -3.3 |
| MOTHE_c03530 | transcriptional regulator KdgR | 385 | 3.718 | -3.3 |
| MOTHE_c17820 | ribonuclease VapC20 | 57 | 555 | -3.2 |
| MOTHE_c20160 | aliphatic sulfonates import ATP-binding protein SsuB | 595 | 5.578 | -3.2 |
| MOTHE_c19840 | aminomethyltransferase | 647 | 5.944 | -3.2 |
| MOTHE_c20100 | anaerobic sulfite reductase subunit C | 2.334 | 20.995 | -3.2 |
| MOTHE_c08840 | manganese transport system membrane protein MntB | 227 | 2.036 | -3.1 |
| MOTHE_c20210 | aliphatic sulfonates import ATP-binding protein SsuB | 9 | 84 | -3.1 |
| MOTHE_c12680 | desulfoferrodoxin | 842 | 8.711 | -3.1 |
| MOTHE_c19830 | glycine cleavage system H protein | 732 | 6.085 | -3.0 |
| MOTHE_c24270 | carbohydrate diacid regulator | 48 | 401 | -3.0 |
| MOTHE_c14370 | methionine synthase | 2.530 | 20.866 | -3.0 |
| MOTHE_c18950 | fused nickel transport protein NikMN | 1.469 | 12.796 | -3.0 |
| MOTHE_c21360 | transcriptional repressor SdpR | 115 | 952 | -3.0 |
| MOTHE_c16140 | transposase | 658 | 4.964 | -2.9 |
| MOTHE_c16090 | transposase | 9 | 80 | -2.9 |
| MOTHE_c21500 | cytochrome bd-I ubiquinol oxidase subunit 2 | 116 | 1.559 | -2.9 |
| MOTHE_c00700 | molybdenum-pterin-binding protein MopA | 217 | 1.582 | -2.8 |
| MOTHE_c02120 | sporulation protein YpeB | 5 | 39 | -2.8 |
| MOTHE_c06280 | ribonuclease VapC20 | 49 | 342 | -2.7 |
| MOTHE_c02410 | putative sigma-54 modulation protein | 1.761 | 11.831 | -2.7 |
| MOTHE_c03840 | HTH-type transcriptional activator CmpR | 805 | 5.256 | -2.7 |
| MOTHE_c24910 | putative beta-barrel protein YwiB | 287 | 1.908 | -2.7 |
| MOTHE_c13870 | fructoselysine 3-epimerase | 584 | 4.133 | -2.7 |
| MOTHE_c19820 | putative glycine dehydrogenase (decarboxylating) subunit 1 | 3.759 | 23.771 | -2.7 |
| MOTHE_c04450 | nitrite reductase [NAD(P)H] | 865 | 6.151 | -2.6 |
| MOTHE_c11810 | putative histidine kinase sensor domain protein | 10 | 70 | -2.6 |
| MOTHE_c19460 | perchlorate reductase subunit alpha precursor | 701 | 4.358 | -2.6 |
| MOTHE_c06400 | ribose transport system permease protein RbsC | 27 | 183 | -2.6 |
| MOTHE_c20220 | putative sulfoacetate--CoA ligase | 50 | 319 | -2.6 |
| MOTHE_c18940 | nickel transport protein NikQ | 1.137 | 7.169 | -2.6 |
| MOTHE_c16560 | tRNA-specific 2-thiouridylase MnmA | 2.581 | 15.664 | -2.6 |
| MOTHE_c05800 | HTH-type transcriptional repressor PurR | 134 | 812 | -2.5 |
| MOTHE_c15760 | accessory gene regulator protein B | 43 | 264 | -2.5 |
| MOTHE_c04720 | putative fluoride ion transporter CrcB | 1.013 | 5.869 | -2.5 |
| MOTHE_c04360 | HTH domain protein | 77 | 439 | -2.5 |
| MOTHE_c00940 | RNA polymerase sigma factor SigX | 39 | 223 | -2.5 |
| MOTHE_c19810 | putative glycine dehydrogenase (decarboxylating) subunit 2 | 4.868 | 26.984 | -2.5 |
| MOTHE_c19310 | carboxymuconolactone decarboxylase family protein | 1.178 | 6.518 | -2.5 |
| MOTHE_c05410 | putative stage IV sporulation protein YqfD | 194 | 1.080 | -2.5 |
| MOTHE_c18930 | nickel import ATP-binding protein NikO | 626 | 3.629 | -2.4 |
| MOTHE_c16310 | selenide. water dikinase | 2.505 | 13.437 | -2.4 |
| MOTHE_c13510 | spore coat protein SA | 3.151 | 16.803 | -2.4 |
| MOTHE_c21370 | immunity protein SdpI | 453 | 2.508 | -2.4 |
| MOTHE_c13800 | nucleotidyltransferase domain protein | 593 | 3.138 | -2.4 |
| MOTHE_c25760 | putative phosphatase YcdX | 735 | 4.081 | -2.4 |
| MOTHE_c14080 | R-phenyllactate dehydratase beta subunit | 4.700 | 25.315 | -2.4 |
| MOTHE_c16550 | PRC-barrel domain protein | 61 | 330 | -2.4 |
| MOTHE_c11540 | transcriptional regulator KdgR | 407 | 2.139 | -2.4 |
| MOTHE_c20240 | putative aliphatic sulfonates transport permease protein SsuC | 10 | 55 | -2.4 |
| MOTHE_c04780 | Lon protease 2 | 664 | 3.430 | -2.4 |
| MOTHE_c20250 | electron transport complex subunit RsxB | 1 | 6 | -2.4 |
| MOTHE_c21250 | HTH-type transcriptional activator TipA | 81 | 417 | -2.3 |
| MOTHE_c21400 | surface layer protein precursor | 5.135 | 26.182 | -2.3 |
| MOTHE_c00920 | spore protein YabP | 7 | 42 | -2.3 |
| MOTHE_c09750 | plasmid stabilization system protein | 3 | 23 | -2.3 |
| MOTHE_c22870 | transcriptional regulator PadR-like family protein | 291 | 1.454 | -2.3 |
| MOTHE_c20850 | ATP phosphoribosyltransferase regulatory subunit | 5.545 | 28.045 | -2.3 |
| MOTHE_c21510 | ATP-binding/permease protein CydD | 207 | 1.751 | -2.3 |
| MOTHE_c02450 | coenzyme PQQ synthesis protein E | 5.672 | 27.980 | -2.3 |
| MOTHE_c02650 | YcfA-like protein | 29 | 144 | -2.3 |
| MOTHE_c18520 | transposase | 522 | 2.544 | -2.3 |
| MOTHE_c20230 | putative aliphatic sulfonates-binding protein precursor | 19 | 101 | -2.3 |
| MOTHE_c12580 | tRNA modification GTPase MnmE | 1.798 | 8.680 | -2.3 |
| MOTHE_c13790 | HEPN domain protein | 587 | 2.831 | -2.2 |
| MOTHE_c00160 | putative N-acetyl-LL-diaminopimelate aminotransferase | 62 | 299 | -2.2 |
| MOTHE_c15770 | flagellar assembly protein H | 124 | 653 | -2.2 |
| MOTHE_c22660 | diflavin flavoprotein A 1 | 2.481 | 11.735 | -2.2 |
| MOTHE_c16700 | thiazole synthase | 432 | 2.040 | -2.2 |
| MOTHE_c01860 | DNA replication and repair protein RecF | 20 | 96 | -2.2 |
| MOTHE_c16690 | sulfur carrier protein ThiS | 10 | 51 | -2.2 |
| MOTHE_c12630 | S-adenosylmethionine decarboxylase proenzyme precursor | 1.423 | 6.680 | -2.2 |
| MOTHE_c12570 | flagellin N-methylase | 671 | 3.099 | -2.2 |
| MOTHE_c01710 | transcriptional regulator CtsR | 605 | 2.810 | -2.2 |
| MOTHE_c14070 | R-phenyllactate dehydratase activator | 1.880 | 8.855 | -2.2 |
| MOTHE_c19450 | tetrathionate reductase subunit B precursor | 141 | 647 | -2.2 |
| MOTHE_c03210 | transposase | 330 | 1.468 | -2.1 |
| MOTHE_c25570 | putative 4-amino-4-deoxy-L-arabinose-phosphoundecaprenol flippase subunit ArnE | 31 | 145 | -2.1 |
| MOTHE_c04110 | phosphate-binding protein PstS 1 precursor | 19.486 | 95.462 | -2.1 |
| MOTHE_c06410 | methylthioribose kinase | 78 | 354 | -2.1 |
| MOTHE_c22290 | acetyl-coenzyme A synthetase | 69 | 309 | -2.1 |
| MOTHE_c17600 | agmatinase | 549 | 2.383 | -2.1 |
| MOTHE_c10940 | response regulator MprA | 389 | 1.693 | -2.1 |
| MOTHE_c18140 | putative prophage phiRv2 integrase | 356 | 1.513 | -2.1 |
| MOTHE_c15570 | nucleotidyltransferase domain protein | 112 | 473 | -2.1 |
| MOTHE_c14360 | R-phenyllactate dehydratase beta subunit | 1.840 | 7.904 | -2.0 |
| MOTHE_c24070 | HTH-type transcriptional repressor NicS | 183 | 751 | -2.0 |
| MOTHE_c19440 | putative hydrogenase 2 b cytochrome subunit | 370 | 1.522 | -2.0 |
| MOTHE_c03160 | integrase core domain protein | 2.386 | 9.705 | -2.0 |
| MOTHE_c16100 | integrase core domain protein | 57 | 234 | -2.0 |
| MOTHE_c26090 | 50S ribosomal protein L34 | 11 | 54 | -2.0 |
| MOTHE_c03500 | endodeoxyribonuclease RusA | 1 | 8 | -2.0 |
| MOTHE_c06130 | protein-export protein SecB | 60 | 243 | -2.0 |
| MOTHE_c20260 | 2-oxoglutarate oxidoreductase subunit KorA | 29 | 123 | -2.0 |
| MOTHE_c06230 | putative transposase. YhgA-like | 591 | 2.336 | -2.0 |
| MOTHE_c18280 | DNA primase | 13 | 53 | -2.0 |
| MOTHE_c06210 | tRNA(fMet)-specific endonuclease VapC | 139 | 556 | -2.0 |
| MOTHE_c00960 | general stress protein 13 | 1.082 | 4.308 | -2.0 |
| MOTHE_c21700 | methionine synthase | 361 | 1.424 | -2.0 |
| MOTHE_c16510 | tRNA/tmRNA (uracil-C(5))-methyltransferase | 1.266 | 4.964 | -2.0 |
| MOTHE_c21220 | putative transposase, YhgA-like | 2.184 | 8.468 | -2.0 |

^1^Mean values of normalized read counts.

**Supplementary table 3: The most upregulated genes of *M. thermoacetica* during growth on CO.**

| **Gene** | **Annotation** | **Substrate** | | **Log2 (fold change)** |
| --- | --- | --- | --- | --- |
|  |  | **CO^1^** | **Glucose^1^** |  |
| MOTHE_c03060 | pyruvate synthase subunit PorD | 424 | 0 | 11.2 |
| MOTHE_c03050 | NADH-dependent phenylglyoxylate dehydrogenase subunit gamma | 1.067 | 1 | 9.6 |
| MOTHE_c07320 | flagellar biosynthetic protein FliQ | 148 | 0 | 9.4 |
| MOTHE_c03070 | pyruvate synthase subunit PorA | 4.887 | 12 | 8.6 |
| MOTHE_c03090 | succinyl-CoA ligase [ADP-forming] subunit alpha | 9.714 | 31 | 8.3 |
| MOTHE_c03080 | NADH-dependent phenylglyoxylate dehydrogenase subunit beta | 2.798 | 9 | 8.2 |
| MOTHE_c03130 | methyl-accepting chemotaxis protein McpC | 908 | 3 | 7.9 |
| MOTHE_c03120 | sodium-dependent dicarboxylate transporter SdcS | 3.284 | 16 | 7.6 |
| MOTHE_c23350 | fructose-1,6-bisphosphatase | 1.559 | 11 | 7.1 |
| MOTHE_c07330 | flagellar biosynthetic protein FliR | 1.437 | 16 | 6.5 |
| MOTHE_c07290 | flagellar motor switch protein FliN | 967 | 11 | 6.4 |
| MOTHE_c06930 | flagellar assembly factor FliW | 783 | 9 | 6.4 |
| MOTHE_c03100 | phenolic acid decarboxylase subunit C | 3.083 | 40 | 6.2 |
| MOTHE_c04690 | primary amine oxidase precursor | 3.746 | 50 | 6.2 |
| MOTHE_c07080 | flagellar protein FlaG | 469 | 7 | 6.0 |
| MOTHE_c07430 | flagellar basal-body rod protein FlgG | 1.414 | 22 | 6.0 |
| MOTHE_c03140 | 5-methyltetrahydrofolate:corrinoid/iron-sulfur protein co-methyltransferase | 741 | 12 | 5.9 |
| MOTHE_c07040 | flagellin C | 46.722 | 765 | 5.9 |
| MOTHE_c07280 | flagellar FliL protein | 742 | 12 | 5.9 |
| MOTHE_c07160 | flagellar M-ring protein | 8.292 | 145 | 5.8 |
| MOTHE_c07170 | flagellar motor switch protein FliG | 4.613 | 80 | 5.8 |
| MOTHE_c07270 | motility protein B | 1.409 | 25 | 5.8 |
| MOTHE_c07150 | flagellar hook-basal body complex protein FliE | 427 | 7 | 5.7 |
| MOTHE_c07180 | Yop proteins translocation protein L | 2.813 | 53 | 5.7 |
| MOTHE_c06910 | endo-1,4-beta-xylanase A precursor | 4.583 | 91 | 5.6 |
| MOTHE_c07100 | flagellar protein FliS | 513 | 10 | 5.6 |
| MOTHE_c07090 | flagellar hook-associated protein 2 | 6.068 | 131 | 5.5 |
| MOTHE_c17520 | motility protein B | 1.052 | 23 | 5.5 |
| MOTHE_c07340 | flagellar biosynthetic protein FlhB | 2.270 | 49 | 5.5 |
| MOTHE_c07140 | flagellar basal-body rod protein FlgC | 1.920 | 43 | 5.5 |
| MOTHE_c23580 | acetylglutamate kinase | 6.038 | 131 | 5.5 |
| MOTHE_c06890 | flagellar hook-associated protein 3 | 1.698 | 38 | 5.4 |
| MOTHE_c07390 | RNA polymerase sigma-D factor | 1.147 | 26 | 5.4 |
| MOTHE_c23590 | arginine biosynthesis bifunctional protein ArgJ | 10.237 | 235 | 5.4 |
| MOTHE_c07220 | basal-body rod modification protein FlgD | 722 | 17 | 5.3 |
| MOTHE_c07420 | flagellar basal-body rod protein FlgG | 1.744 | 45 | 5.2 |
| MOTHE_c00150 | phosphoenolpyruvate-protein phosphotransferase | 19.372 | 453 | 5.2 |
| MOTHE_c07200 | flagellar FliJ protein | 924 | 24 | 5.2 |
| MOTHE_c00140 | phosphocarrier protein HPr | 612 | 14 | 5.2 |
| MOTHE_c23600 | N-acetyl-gamma-glutamyl-phosphate reductase | 8.585 | 235 | 5.2 |
| MOTHE_c06960 | putative pyridoxal phosphate-dependent aminotransferase EpsN | 1.569 | 46 | 5.1 |
| MOTHE_c07240 | flagellar basal-body rod protein FlgG | 2.900 | 84 | 5.1 |
| MOTHE_c17510 | chemotaxis protein PomA | 753 | 22 | 5.1 |
| MOTHE_c07210 | flagellar hook-length control protein FliK | 10.940 | 325 | 5.1 |
| MOTHE_c07300 | flagellar biosynthesis protein FliO | 650 | 19 | 5.0 |
| MOTHE_c07350 | flagellar biosynthesis protein FlhA | 4.598 | 147 | 5.0 |
| MOTHE_c07310 | flagellar biosynthetic protein FliP precursor | 3.158 | 101 | 4.9 |
| MOTHE_c07410 | YceG-like family protein | 830 | 26 | 4.9 |
| MOTHE_c07130 | flagellar basal body rod protein FlgB | 1.306 | 43 | 4.9 |
| MOTHE_c07260 | chemotaxis protein PomA | 2.256 | 74 | 4.9 |
| MOTHE_c07190 | putative ATP synthase YscN | 9.253 | 314 | 4.9 |
| MOTHE_c07370 | flagellum site-determining protein YlxH | 2.163 | 78 | 4.8 |
| MOTHE_c00120 | PTS system fructose-specific EIIBC component | 17.635 | 549 | 4.8 |
| MOTHE_c00130 | PTS system fructose-specific EIIABC component | 3.654 | 119 | 4.7 |
| MOTHE_c06880 | flagellar hook-associated protein 1 | 3.546 | 135 | 4.7 |
| MOTHE_c21630 | branched-chain-amino-acid aminotransferase | 2.387 | 92 | 4.7 |
| MOTHE_c23570 | acetylornithine aminotransferase | 11.061 | 447 | 4.6 |
| MOTHE_c03110 | phenolic acid decarboxylase subunit D | 82 | 3 | 4.6 |
| MOTHE_c07360 | flagellar biosynthesis protein FlhF | 3.649 | 164 | 4.5 |
| MOTHE_c22530 | formate dehydrogenase H | 10 | 0 | 4.4 |
| MOTHE_c07380 | flagellar brake protein YcgR | 1.753 | 85 | 4.3 |
| MOTHE_c06870 | FlgN protein | 1.278 | 62 | 4.3 |
| MOTHE_c09600 | flagellar biosynthetic protein FlhB | 9 | 0 | 4.3 |
| MOTHE_c08240 | bifunctional protein PyrR | 4.595 | 225 | 4.3 |
| MOTHE_c06950 | UDP-glucose 4-epimerase | 1.796 | 93 | 4.3 |
| MOTHE_c07250 | flagellar protein (FlbD) | 184 | 10 | 4.2 |
| MOTHE_c03170 | methylcobalamin:coenzyme M methyltransferase | 109 | 6 | 4.2 |
| MOTHE_c08250 | aspartate carbamoyltransferase | 6.405 | 336 | 4.1 |
| MOTHE_c03040 | transcriptional regulator KdgR | 1.202 | 70 | 4.1 |
| MOTHE_c22520 | formate dehydrogenase H | 118 | 6 | 4.1 |
| MOTHE_c21620 | aminodeoxychorismate synthase component 1 | 6.209 | 366 | 4.1 |
| MOTHE_c06970 | phosphoribosylamine--glycine ligase | 437 | 27 | 3.9 |
| MOTHE_c08260 | dihydroorotase | 8.738 | 528 | 3.9 |
| MOTHE_c06860 | anti-sigma-28 factor. FlgM | 731 | 50 | 3.8 |
| MOTHE_c15860 | UDP-glucose 4-epimerase | 337 | 24 | 3.8 |
| MOTHE_c06980 | phosphoglycolate phosphatase | 49 | 3 | 3.7 |
| MOTHE_c25510 | 50S ribosomal protein L10 | 5.032 | 381 | 3.7 |
| MOTHE_c00110 | tagatose-6-phosphate kinase | 3.519 | 232 | 3.6 |
| MOTHE_c10870 | putative endonuclease 4 | 250 | 21 | 3.5 |
| MOTHE_c15890 | pyruvate synthase subunit PorB | 38.200 | 3.245 | 3.5 |
| MOTHE_c06030 | teichoic acids export ATP-binding protein TagH | 143 | 12 | 3.5 |
| MOTHE_c07120 | SpoVT / AbrB like domain protein | 66 | 5 | 3.5 |
| MOTHE_c25400 | 50S ribosomal protein L4 | 6.282 | 581 | 3.4 |
| MOTHE_c05260 | stage II sporulation protein P (SpoIIP) | 168 | 15 | 3.4 |
| MOTHE_c25380 | 50S ribosomal protein L2 | 9.526 | 911 | 3.4 |
| MOTHE_c19190 | NADP^+^-reducing hydrogenase subunit HydD | 5 | 0 | 3.4 |
| MOTHE_c25410 | 50S ribosomal protein L3 | 10.333 | 1.041 | 3.3 |
| MOTHE_c25440 | elongation factor G | 45.280 | 4.596 | 3.3 |
| MOTHE_c25310 | 50S ribosomal protein L14 | 1.442 | 146 | 3.3 |
| MOTHE_c06990 | GDP/UDP-N,N'-diacetylbacillosamine 2-epimerase (hydrolyzing) | 56 | 5 | 3.3 |
| MOTHE_c25500 | 50S ribosomal protein L7/L12 | 3.705 | 380 | 3.3 |
| MOTHE_c15900 | NADH-dependent phenylglyoxylate dehydrogenase subunit alpha | 47.357 | 4.887 | 3.2 |
| MOTHE_c25270 | 30S ribosomal protein S8 | 1.102 | 112 | 3.2 |
| MOTHE_c25320 | 30S ribosomal protein S17 | 651 | 67 | 3.2 |
| MOTHE_c25350 | 30S ribosomal protein S3 | 6.951 | 754 | 3.2 |
| MOTHE_c25360 | 50S ribosomal protein L22 | 3.872 | 423 | 3.2 |
| MOTHE_c15240 | stage III sporulation protein AE precursor | 63 | 7 | 3.2 |
| MOTHE_c15910 | pyruvate synthase subunit PorD | 27.358 | 2.948 | 3.2 |
| MOTHE_c25290 | 50S ribosomal protein L5 | 4.217 | 477 | 3.1 |
| MOTHE_c22540 | putative formate transporter 1 | 1.936 | 220 | 3.1 |
| MOTHE_c20990 | phosphoribosylaminoimidazole-succinocarboxamide synthase | 3.507 | 400 | 3.1 |
| MOTHE_c25370 | 30S ribosomal protein S19 | 1.060 | 122 | 3.1 |
| MOTHE_c20900 | spore germination protein B1 | 881 | 102 | 3.1 |
| MOTHE_c03190 | methionine synthase | 48 | 4 | 3.1 |
| MOTHE_c25390 | 50S ribosomal protein L23 | 1.846 | 219 | 3.1 |
| MOTHE_c25300 | 50S ribosomal protein L24 | 1.653 | 197 | 3.1 |
| MOTHE_c15870 | oxalate:formate antiporter | 101 | 11 | 3.0 |
| MOTHE_c05610 | tagatose-6-phosphate kinase | 483 | 55 | 3.0 |
| MOTHE_c25250 | 50S ribosomal protein L18 | 2.558 | 316 | 3.0 |
| MOTHE_c10670 | EamA-like transporter family protein | 216 | 26 | 3.0 |
| MOTHE_c25430 | elongation factor Tu-B | 37.262 | 4.707 | 3.0 |
| MOTHE_c20890 | spore germination protein YndE | 215 | 27 | 3.0 |
| MOTHE_c17290 | multifunctional cyclase-dehydratase-3-O-methyl transferase TcmN | 866 | 108 | 3.0 |
| MOTHE_c07500 | flagellar motor switch protein FliN | 2.810 | 362 | 2.9 |
| MOTHE_c05160 | citrate transporter | 4.291 | 556 | 2.9 |
| MOTHE_c25420 | 30S ribosomal protein S10 | 2.424 | 316 | 2.9 |
| MOTHE_c25340 | 50S ribosomal protein L16 | 3.218 | 423 | 2.9 |
| MOTHE_c02200 | OPT oligopeptide transporter protein | 36.359 | 4.829 | 2.9 |
| MOTHE_c25330 | 50S ribosomal protein L29 | 397 | 51 | 2.9 |
| MOTHE_c07490 | flagellar motor switch protein FliM | 2.020 | 279 | 2.8 |
| MOTHE_c13440 | putative spore protein YtfJ | 130 | 17 | 2.8 |
| MOTHE_c25260 | 50S ribosomal protein L6 | 4.424 | 614 | 2.8 |
| MOTHE_c05130 | putative nicotinate-nucleotide adenylyltransferase | 3.677 | 521 | 2.8 |
| MOTHE_c25240 | 30S ribosomal protein S5 | 4.739 | 672 | 2.8 |
| MOTHE_c23850 | ferredoxin--NADP reductase | 3.173 | 455 | 2.8 |
| MOTHE_c05150 | leucine--tRNA ligase | 18.408 | 2.682 | 2.8 |
| MOTHE_c06040 | putative teichuronic acid biosynthesis glycosyltransferase TuaH | 92 | 13 | 2.8 |
| MOTHE_c09570 | enterobactin exporter EntS | 296 | 42 | 2.8 |
| MOTHE_c10910 | glutamine transport ATP-binding protein GlnQ | 2.356 | 346 | 2.8 |
| MOTHE_c07970 | sporulation sigma-E factor-processing peptidase | 44 | 6 | 2.7 |
| MOTHE_c18420 | PhoU domain protein | 231 | 34 | 2.7 |
| MOTHE_c20950 | amidophosphoribosyltransferase precursor | 4.817 | 739 | 2.7 |
| MOTHE_c21560 | uroporphyrinogen decarboxylase | 129 | 19 | 2.7 |
| MOTHE_c07480 | chemotaxis protein CheY | 565 | 90 | 2.6 |
| MOTHE_c19260 | endonuclease III | 622 | 98 | 2.6 |
| MOTHE_c04960 | putative zinc metalloprotease Rip3 | 491 | 78 | 2.6 |
| MOTHE_c07470 | CheY-P phosphatase CheC | 859 | 138 | 2.6 |
| MOTHE_c25280 | 30S ribosomal protein S14 type Z | 264 | 40 | 2.6 |
| MOTHE_c12760 | glutamate synthase [NADPH] large chain | 1.232 | 199 | 2.6 |
| MOTHE_c25490 | DNA-directed RNA polymerase subunit beta | 16.773 | 2.752 | 2.6 |
| MOTHE_c05140 | ribosomal silencing factor RsfS | 1.056 | 173 | 2.6 |
| MOTHE_c10410 | ribonuclease Y | 6.999 | 1.152 | 2.6 |
| MOTHE_c25150 | 30S ribosomal protein S13 | 1.971 | 328 | 2.6 |
| MOTHE_c10640 | threonine-phosphate decarboxylase | 1.086 | 182 | 2.6 |
| MOTHE_c17310 | phosphomethylpyrimidine synthase | 957 | 159 | 2.6 |
| MOTHE_c20930 | phosphoribosylglycinamide formyltransferase | 1.367 | 230 | 2.6 |
| MOTHE_c22290 | acetyl-coenzyme A synthetase | 1.837 | 309 | 2.5 |
| MOTHE_c25450 | 30S ribosomal protein S7 | 10.376 | 1.770 | 2.5 |
| MOTHE_c08290 | orotidine 5'-phosphate decarboxylase | 1.834 | 278 | 2.5 |
| MOTHE_c20600 | spore coat protein F precursor | 213 | 35 | 2.5 |
| MOTHE_c05620 | D-ribose-binding periplasmic protein precursor | 583 | 95 | 2.5 |
| MOTHE_c13320 | universal stress protein family protein | 196 | 33 | 2.5 |
| MOTHE_c17300 | nicotinate-nucleotide--dimethylbenzimidazole phosphoribosyltransferase | 1.176 | 209 | 2.5 |
| MOTHE_c25190 | methionine aminopeptidase 1 | 6.472 | 1.168 | 2.5 |
| MOTHE_c02940 | radical SAM superfamily protein | 1.701 | 310 | 2.4 |
| MOTHE_c23970 | macrolide export protein MacA | 770 | 137 | 2.4 |
| MOTHE_c15880 | oxalate:formate antiporter | 770 | 139 | 2.4 |
| MOTHE_c23490 | potassium/proton antiporter | 20 | 3 | 2.4 |
| MOTHE_c09560 | multidrug resistance protein 3 | 428 | 78 | 2.4 |
| MOTHE_c09430 | NAD(P)H-quinone oxidoreductase chain 4 1 | 7.911 | 1.452 | 2.4 |
| MOTHE_c10900 | inner membrane amino-acid ABC transporter permease protein YecS | 2.380 | 440 | 2.4 |
| MOTHE_c02550 | chitodextrinase precursor | 1.222 | 227 | 2.4 |
| MOTHE_c21000 | adenylosuccinate lyase | 8.751 | 1.633 | 2.4 |
| MOTHE_c20960 | phosphoribosylformylglycinamidine synthase 2 | 8.823 | 1.650 | 2.4 |
| MOTHE_c00350 | sigmaK-factor processing regulatory protein BofA | 46 | 8 | 2.4 |
| MOTHE_c21010 | N5-carboxyaminoimidazole ribonucleotide mutase | 3.226 | 608 | 2.4 |
| MOTHE_c10630 | L-threonine kinase | 1.358 | 256 | 2.4 |
| MOTHE_c12770 | glutamine synthetase | 2.658 | 503 | 2.4 |
| MOTHE_c02250 | 2,5-dihydroxypyridine 5,6-dioxygenase | 14.925 | 2.839 | 2.4 |
| MOTHE_c10010 | regulator of sigma-W protease RasP | 5.150 | 991 | 2.4 |
| MOTHE_c25460 | 30S ribosomal protein S12 | 10.192 | 1.973 | 2.4 |
| MOTHE_c17320 | phosphomethylpyrimidine synthase | 774 | 145 | 2.3 |
| MOTHE_c15930 | ATP-dependent zinc metalloprotease FtsH | 9.018 | 1.737 | 2.3 |
| MOTHE_c08710 | 6,7-dimethyl-8-ribityllumazine synthase | 1.283 | 251 | 2.3 |
| MOTHE_c24880 | lipid II:glycine glycyltransferase | 2.439 | 483 | 2.3 |
| MOTHE_c25140 | 30S ribosomal protein S11 | 1.988 | 396 | 2.3 |
| MOTHE_c25170 | translation initiation factor IF-1 | 422 | 83 | 2.3 |
| MOTHE_c20640 | xylose transport system permease protein XylH | 5.947 | 1.197 | 2.3 |
| MOTHE_c10040 | DNA polymerase III PolC-type | 8.352 | 1.697 | 2.3 |
| MOTHE_c14770 | methyl-accepting chemotaxis protein McpA | 718 | 144 | 2.3 |
| MOTHE_c09440 | NADH-quinone oxidoreductase subunit N | 8.006 | 1.620 | 2.3 |
| MOTHE_c13330 | Trk system potassium uptake protein TrkA | 148 | 29 | 2.3 |
| MOTHE_c22410 | hydrogenase 3 maturation protease | 43 | 8 | 2.3 |
| MOTHE_c19380 | putative ABC transporter ATP-binding protein YbhF | 43 | 8 | 2.2 |
| MOTHE_c10030 | proline--tRNA ligase | 10.563 | 2.223 | 2.2 |
| MOTHE_c13130 | isopentenyl-diphosphate delta-isomerase | 2.329 | 490 | 2.2 |
| MOTHE_c20980 | phosphoribosylformylglycinamidine synthase subunit PurS | 527 | 111 | 2.2 |
| MOTHE_c23520 | fumarate hydratase class I. anaerobic | 3.600 | 748 | 2.2 |
| MOTHE_c20880 | spore germination protein B3 precursor | 291 | 60 | 2.2 |
| MOTHE_c25210 | protein translocase subunit SecY | 10.732 | 2.310 | 2.2 |
| MOTHE_c23190 | 2,3-dimethylmalate dehydratase small subunit | 1.752 | 378 | 2.2 |
| MOTHE_c10130 | riboflavin biosynthesis protein RibF | 1.318 | 283 | 2.2 |
| MOTHE_c10180 | D-alanyl-D-alanine carboxypeptidase DacF precursor | 609 | 132 | 2.2 |
| MOTHE_c22060 | response regulator PleD | 756 | 162 | 2.2 |
| MOTHE_c10590 | cobalamin biosynthesis protein CbiB | 2.310 | 509 | 2.2 |
| MOTHE_c13340 | Trk system potassium uptake protein TrkA | 305 | 67 | 2.2 |
| MOTHE_c19250 | ferredoxin-1 | 214 | 47 | 2.2 |
| MOTHE_c13110 | stage II sporulation protein P (SpoIIP) | 104 | 22 | 2.2 |
| MOTHE_c25230 | 50S ribosomal protein L30 | 214 | 46 | 2.2 |
| MOTHE_c25850 | leucine/isoleucine/valine transporter permease subunit | 2.824 | 634 | 2.2 |
| MOTHE_c20970 | phosphoribosylformylglycinamidine synthase 1 | 2.912 | 654 | 2.1 |
| MOTHE_c25950 | putative phosphoribosyl transferasec | 629 | 140 | 2.1 |
| MOTHE_c13590 | SpoVA protein | 4 | 0 | 2.1 |
| MOTHE_c09420 | NADH-quinone oxidoreductase subunit 12 | 7.707 | 1.765 | 2.1 |
| MOTHE_c12400 | acetate CoA-transferase YdiF | 45 | 10 | 2.1 |
| MOTHE_c23210 | 2,3-dimethylmalate dehydratase large subunit | 1.087 | 251 | 2.1 |
| MOTHE_c22470 | hydrogenase-4 component B | 50 | 11 | 2.1 |
| MOTHE_c25220 | 50S ribosomal protein L15 | 3.067 | 712 | 2.1 |
| MOTHE_c13300 | putative amino acid permease YhdG | 1.186 | 278 | 2.1 |
| MOTHE_c02580 | cellulosome-anchoring protein precursor | 137 | 31 | 2.1 |
| MOTHE_c07440 | TPR repeat-containing protein YrrB | 9 | 1 | 2.1 |
| MOTHE_c23980 | putative multidrug resistance protein EmrK | 391 | 91 | 2.0 |
| MOTHE_c15200 | phenylalanine-specific permease | 61 | 14 | 2.0 |
| MOTHE_c01680 | serine/threonine exchanger SteT | 3.507 | 856 | 2.0 |
| MOTHE_c12740 | iron-sulfur protein | 512 | 123 | 2.0 |
| MOTHE_c23200 | 2,3-dimethylmalate dehydratase large subunit | 1.840 | 447 | 2.0 |
| MOTHE_c04950 | stage II sporulation protein Q | 790 | 192 | 2.0 |
| MOTHE_c22510 | hydrogenase-4 component A | 22 | 4 | 2.0 |
| MOTHE_c25080 | energy-coupling factor transporter transmembrane protein EcfT | 4.262 | 1.042 | 2.0 |
| MOTHE_c25130 | 30S ribosomal protein S4 | 4.097 | 1.001 | 2.0 |
| MOTHE_c10880 | EamA-like transporter family protein | 404 | 100 | 2.0 |
| MOTHE_c20650 | xylose import ATP-binding protein XylG | 10.294 | 2.539 | 2.0 |
| MOTHE_c17740 | YtxC-like family protein | 126 | 29 | 2.0 |
| MOTHE_c17670 | Ktr system potassium uptake protein A | 1.810 | 450 | 2.0 |
| MOTHE_c00540 | cell wall-binding protein YocH precursor | 39 | 8 | 2.0 |
| MOTHE_c02270 | colanic acid biosynthesis protein | 5.225 | 1.302 | 2.0 |
| MOTHE_c05210 | sodium bile acid symporter family protein | 1.971 | 494 | 2.0 |
| MOTHE_c12750 | glutamate synthase [NADPH] large chain precursor | 1.688 | 421 | 2.0 |
| MOTHE_c12880 | aspartokinase | 7.387 | 1.885 | 2.0 |
| MOTHE_c25840 | lipopolysaccharide export system ATP-binding protein LptB | 2.195 | 561 | 2.0 |
| MOTHE_c23550 | argininosuccinate synthase | 13.295 | 3.327 | 2.0 |
| MOTHE_c05200 | ribosomal protein L11 methyltransferase | 2.463 | 630 | 2.0 |

^1^Mean values of normalized read counts.

**Supplementary table 4: The most downregulated genes of *M. thermoacetica* during growth on CO.**

| **Gene** | **Annotation** | **substrate** | | **Log2 (fold change)** |
| --- | --- | --- | --- | --- |
|  |  | **CO^1^** | **Glucose^1^** |  |
| MOTHE_c14240 | ferrous iron transport protein A | 1 | 1.929 | -10.5 |
| MOTHE_c14230 | FeoA domain protein | 2 | 2.641 | -10.2 |
| MOTHE_c14220 | ferrous iron transport protein B | 77 | 52.267 | -9.3 |
| MOTHE_c11590 | FeoA domain protein | 39 | 16.978 | -8.7 |
| MOTHE_c14210 | cyclic di-GMP phosphodiesterase response regulator RpfG | 43 | 8.616 | -7.6 |
| MOTHE_c04090 | Hsp20/alpha crystallin family protein | 34 | 5.233 | -7.2 |
| MOTHE_c11580 | ferrous iron transport protein B | 876 | 123.223 | -7.1 |
| MOTHE_c11280 | alkaline phosphatase precursor | 1.444 | 203.815 | -7.0 |
| MOTHE_c03560 | KHG/KDPG aldolase | 119 | 15.443 | -7.0 |
| MOTHE_c23830 | putative FAD-linked oxidoreductase | 544 | 58.014 | -6.7 |
| MOTHE_c04100 | calcium-transporting ATPase | 5 | 535 | -6.6 |
| MOTHE_c03550 | 2-dehydro-3-deoxygluconokinase | 239 | 21.736 | -6.5 |
| MOTHE_c17280 | Electron bifurcating hydrogenase subunit HydC | 23 | 2.493 | -6.4 |
| MOTHE_c17270 | Electron bifurcating hydrogenase subunit HydB | 196 | 17.444 | -6.3 |
| MOTHE_c03540 | K^+^-stimulated pyrophosphate-energized sodium pump | 313 | 23.805 | -6.2 |
| MOTHE_c23820 | anaerobic glycerol-3-phosphate dehydrogenase subunit C | 543 | 39.923 | -6.2 |
| MOTHE_c19460 | perchlorate reductase subunit alpha precursor | 61 | 4.358 | -6.1 |
| MOTHE_c17260 | Electron bifurcating hydrogenase subunit HydA | 229 | 16.489 | -6.0 |
| MOTHE_c23810 | lactate utilization protein C | 196 | 11.556 | -5.9 |
| MOTHE_c16600 | HTH-type transcriptional regulator CymR | 391 | 22.431 | -5.8 |
| MOTHE_c19450 | tetrathionate reductase subunit B precursor | 14 | 647 | -5.5 |
| MOTHE_c16590 | cysteine desulfurase IscS | 3.479 | 145.065 | -5.3 |
| MOTHE_c16580 | iron-sulfur cluster assembly scaffold protein IscU | 497 | 19.403 | -5.2 |
| MOTHE_c13870 | fructoselysine 3-epimerase | 107 | 4.133 | -5.2 |
| MOTHE_c23800 | lactate utilization protein B | 1.157 | 38.069 | -5.0 |
| MOTHE_c15680 | primary amine oxidase precursor | 7 | 228 | -5.0 |
| MOTHE_c19440 | putative hydrogenase 2 b cytochrome subunit | 47 | 1.522 | -5.0 |
| MOTHE_c02130 | spore cortex-lytic enzyme precursor | 0 | 15 | -4.9 |
| MOTHE_c20120 | O-acetylserine sulfhydrylase | 1.020 | 28.558 | -4.8 |
| MOTHE_c06370 | glucitol operon repressor | 42 | 1.119 | -4.7 |
| MOTHE_c23790 | HTH-type transcriptional regulator LutR | 508 | 13.263 | -4.7 |
| MOTHE_c08870 | peroxide-responsive repressor PerR | 227 | 5.039 | -4.4 |
| MOTHE_c08860 | high-affinity zinc uptake system binding-protein ZnuA precursor | 625 | 13.775 | -4.4 |
| MOTHE_c14200 | rhodocoxin reductase | 573 | 12.812 | -4.4 |
| MOTHE_c20110 | putative adenylyltransferase/sulfurtransferase MoeZ | 2.575 | 51.540 | -4.3 |
| MOTHE_c17130 | cysteine synthase | 710 | 14.251 | -4.3 |
| MOTHE_c20080 | sulfur carrier protein ThiS | 73 | 1.405 | -4.2 |
| MOTHE_c23780 | glycolate permease GlcA | 1.065 | 19.355 | -4.2 |
| MOTHE_c08850 | hemin import ATP-binding protein HmuV | 560 | 9.982 | -4.1 |
| MOTHE_c20130 | carbon monoxide dehydrogenase 1 | 2.195 | 39.201 | -4.1 |
| MOTHE_c04110 | phosphate-binding protein PstS 1 precursor | 5.426 | 95.462 | -4.0 |
| MOTHE_c15700 | nucleotidyltransferase domain protein | 4 | 66 | -3.9 |
| MOTHE_c20100 | anaerobic sulfite reductase subunit C | 1.603 | 20.995 | -3.7 |
| MOTHE_c20150 | bicarbonate transport system permease protein CmpB | 372 | 4.529 | -3.6 |
| MOTHE_c14730 | sn-glycerol-3-phosphate transport system permease protein UgpA | 106 | 1.467 | -3.6 |
| MOTHE_c14710 | sn-glycerol-3-phosphate import ATP-binding protein UgpC | 171 | 2.236 | -3.5 |
| MOTHE_c06670 | 4-hydroxythreonine-4-phosphate dehydrogenase 2 | 143 | 1.651 | -3.5 |
| MOTHE_c08840 | manganese transport system membrane protein MntB | 182 | 2.036 | -3.5 |
| MOTHE_c21980 | transposase | 108 | 1.745 | -3.4 |
| MOTHE_c14160 | pyridoxamine 5'-phosphate oxidase | 58 | 665 | -3.4 |
| MOTHE_c20160 | aliphatic sulfonates import ATP-binding protein SsuB | 513 | 5.578 | -3.4 |
| MOTHE_c20850 | ATP phosphoribosyltransferase regulatory subunit | 2.605 | 28.045 | -3.4 |
| MOTHE_c20140 | putative aliphatic sulfonates-binding protein precursor | 659 | 6.975 | -3.4 |
| MOTHE_c06380 | D-allose-binding periplasmic protein precursor | 117 | 1.189 | -3.3 |
| MOTHE_c06680 | Gnt-II system L-idonate transporter | 90 | 857 | -3.2 |
| MOTHE_c21360 | transcriptional repressor SdpR | 98 | 952 | -3.2 |
| MOTHE_c03530 | transcriptional regulator KdgR | 404 | 3.718 | -3.2 |
| MOTHE_c03840 | HTH-type transcriptional activator CmpR | 596 | 5.256 | -3.1 |
| MOTHE_c22300 | sensor histidine kinase YehU | 537 | 4.682 | -3.1 |
| MOTHE_c14720 | L-arabinose transport system permease protein AraQ | 84 | 854 | -3.1 |
| MOTHE_c02410 | putative sigma-54 modulation protein | 1.380 | 11.831 | -3.1 |
| MOTHE_c14190 | ribonuclease BN | 643 | 5.754 | -3.1 |
| MOTHE_c15570 | nucleotidyltransferase domain protein | 55 | 473 | -3.1 |
| MOTHE_c02120 | sporulation protein YpeB | 4 | 39 | -3.1 |
| MOTHE_c24910 | putative beta-barrel protein YwiB | 223 | 1.908 | -3.1 |
| MOTHE_c22310 | sensory transduction protein LytR | 195 | 1.637 | -3.1 |
| MOTHE_c06280 | ribonuclease VapC20 | 42 | 342 | -3.0 |
| MOTHE_c02530 | HTH-type transcriptional regulator ImmR | 36 | 291 | -3.0 |
| MOTHE_c11810 | putative histidine kinase sensor domain protein | 8 | 70 | -3.0 |
| MOTHE_c00700 | molybdenum-pterin-binding protein MopA | 197 | 1.582 | -3.0 |
| MOTHE_c16560 | tRNA-specific 2-thiouridylase MnmA | 1.942 | 15.664 | -3.0 |
| MOTHE_c06390 | ribose import ATP-binding protein RbsA | 50 | 410 | -2.9 |
| MOTHE_c01710 | transcriptional regulator CtsR | 373 | 2.810 | -2.9 |
| MOTHE_c17600 | agmatinase | 326 | 2.383 | -2.9 |
| MOTHE_c13790 | HEPN domain protein | 391 | 2.831 | -2.8 |
| MOTHE_c16150 | RNA polymerase sigma factor RpoD | 44 | 318 | -2.8 |
| MOTHE_c23300 | zinc ribbon domain protein | 20 | 147 | -2.8 |
| MOTHE_c13800 | nucleotidyltransferase domain protein | 461 | 3.138 | -2.8 |
| MOTHE_c17820 | ribonuclease VapC20 | 81 | 555 | -2.7 |
| MOTHE_c18900 | MarR family protein | 98 | 669 | -2.7 |
| MOTHE_c18140 | putative prophage phiRv2 integrase | 224 | 1.513 | -2.7 |
| MOTHE_c16550 | PRC-barrel domain protein | 48 | 330 | -2.7 |
| MOTHE_c21430 | Phd_YefM | 9 | 63 | -2.7 |
| MOTHE_c22870 | transcriptional regulator PadR-like family protein | 226 | 1.454 | -2.7 |
| MOTHE_c12670 | high molecular weight rubredoxin | 121 | 962 | -2.7 |
| MOTHE_c16690 | sulfur carrier protein ThiS | 7 | 51 | -2.7 |
| MOTHE_c13510 | spore coat protein SA | 2.672 | 16.803 | -2.6 |
| MOTHE_c18520 | transposase | 401 | 2.544 | -2.6 |
| MOTHE_c03210 | transposase | 235 | 1.468 | -2.6 |
| MOTHE_c05900 | integrase core domain protein | 30 | 208 | -2.6 |
| MOTHE_c14120 | Fe^3+^-citrate-binding protein YfmC precursor | 2 | 12 | -2.6 |
| MOTHE_c11540 | transcriptional regulator KdgR | 355 | 2.139 | -2.6 |
| MOTHE_c21370 | immunity protein SdpI | 417 | 2.508 | -2.5 |
| MOTHE_c04720 | putative fluoride ion transporter CrcB | 998 | 5.869 | -2.5 |
| MOTHE_c20780 | phosphoribosyl-ATP pyrophosphatase | 1.183 | 6.737 | -2.5 |
| MOTHE_c21220 | putative transposase. YhgA-like | 1.521 | 8.468 | -2.5 |
| MOTHE_c20820 | imidazoleglycerol-phosphate dehydratase | 1.263 | 7.104 | -2.5 |
| MOTHE_c04780 | Lon protease 2 | 621 | 3.430 | -2.5 |
| MOTHE_c20210 | aliphatic sulfonates import ATP-binding protein SsuB | 13 | 84 | -2.4 |
| MOTHE_c24030 | multidrug resistance protein MdtN | 34 | 202 | -2.4 |
| MOTHE_c16090 | transposase | 13 | 80 | -2.4 |
| MOTHE_c14290 | ATP-dependent (S)-NAD(P)H-hydrate dehydratase | 2.556 | 15.452 | -2.4 |
| MOTHE_c12580 | tRNA modification GTPase MnmE | 1.613 | 8.680 | -2.4 |
| MOTHE_c00160 | putative N-acetyl-LL-diaminopimelate aminotransferase | 55 | 299 | -2.4 |
| MOTHE_c04360 | HTH domain protein | 83 | 439 | -2.4 |
| MOTHE_c20830 | histidinol dehydrogenase | 2.804 | 14.907 | -2.4 |
| MOTHE_c05800 | HTH-type transcriptional repressor PurR | 150 | 812 | -2.4 |
| MOTHE_c14410 | succinate dehydrogenase/fumarate reductase iron-sulfur subunit | 6.252 | 33.637 | -2.4 |
| MOTHE_c09470 | acetophenone carboxylase alpha subunit | 2.588 | 13.600 | -2.4 |
| MOTHE_c19310 | carboxymuconolactone decarboxylase family protein | 1.244 | 6.518 | -2.4 |
| MOTHE_c12570 | flagellin N-methylase | 591 | 3.099 | -2.4 |
| MOTHE_c20810 | imidazole glycerol phosphate synthase subunit HisH 1 | 733 | 3.833 | -2.4 |
| MOTHE_c19830 | glycine cleavage system H protein | 1.177 | 6.085 | -2.4 |
| MOTHE_c24270 | carbohydrate diacid regulator | 78 | 401 | -2.3 |
| MOTHE_c24070 | HTH-type transcriptional repressor NicS | 146 | 751 | -2.3 |
| MOTHE_c15560 | HEPN domain protein | 69 | 355 | -2.3 |
| MOTHE_c21250 | HTH-type transcriptional activator TipA | 82 | 417 | -2.3 |
| MOTHE_c14040 | serine dehydratase alpha chain | 106 | 546 | -2.3 |
| MOTHE_c06210 | tRNA(fMet)-specific endonuclease VapC | 111 | 556 | -2.3 |
| MOTHE_c20790 | imidazole glycerol phosphate synthase subunit HisF | 1.331 | 6.549 | -2.3 |
| MOTHE_c21400 | surface layer protein precursor | 5.422 | 26.182 | -2.3 |
| MOTHE_c17650 | apolipoprotein A1/A4/E domain protein | 984 | 4.716 | -2.3 |
| MOTHE_c21190 | PIN domain protein | 427 | 2.063 | -2.2 |
| MOTHE_c09750 | plasmid stabilization system protein | 3 | 23 | -2.2 |
| MOTHE_c11000 | inosine-5'-monophosphate dehydrogenase | 499 | 2.365 | -2.2 |
| MOTHE_c21700 | methionine synthase | 298 | 1.424 | -2.2 |
| MOTHE_c22660 | diflavin flavoprotein A 1 | 2.464 | 11.735 | -2.2 |
| MOTHE_c13850 | nucleotidyltransferase domain protein | 212 | 1.008 | -2.2 |
| MOTHE_c06230 | putative transposase, YhgA-like | 496 | 2.336 | -2.2 |
| MOTHE_c20840 | ATP phosphoribosyltransferase | 2.263 | 10.677 | -2.2 |
| MOTHE_c12040 | nicotinate dehydrogenase large molybdopterin subunit | 416 | 1.941 | -2.2 |
| MOTHE_c05480 | cytidine deaminase | 1.452 | 6.774 | -2.2 |
| MOTHE_c03680 | biofilm growth-associated repressor | 6 | 35 | -2.2 |
| MOTHE_c10940 | response regulator MprA | 360 | 1.693 | -2.2 |
| MOTHE_c13830 | RNA polymerase-associated protein RapA | 2 | 14 | -2.2 |
| MOTHE_c12630 | S-adenosylmethionine decarboxylase proenzyme precursor | 1.434 | 6.680 | -2.2 |
| MOTHE_c16780 | EcoKI restriction-modification system protein HsdS | 99 | 453 | -2.2 |
| MOTHE_c01860 | DNA replication and repair protein RecF | 20 | 96 | -2.2 |
| MOTHE_c17720 | threonine--tRNA ligase 2 | 2.415 | 10.984 | -2.2 |
| MOTHE_c24040 | putative ABC transporter ATP-binding protein YbhF | 28 | 136 | -2.2 |
| MOTHE_c20800 | 1-(5-phosphoribosyl)-5-[(5-phosphoribosylamino)methylideneamino] imidazole-4-carboxamide isomerase | 1.255 | 5.740 | -2.2 |
| MOTHE_c15690 | HEPN domain protein | 26 | 124 | -2.2 |
| MOTHE_c16100 | integrase core domain protein | 51 | 234 | -2.2 |
| MOTHE_c25760 | putative phosphatase YcdX | 863 | 4.081 | -2.2 |
| MOTHE_c22840 | major facilitator superfamily protein | 0 | 5 | -2.1 |
| MOTHE_c15950 | zinc ribbon domain protein | 248 | 1.111 | -2.1 |
| MOTHE_c18950 | fused nickel transport protein NikMN | 2.613 | 12.796 | -2.1 |
| MOTHE_c14420 | ferredoxin--NADP reductase | 29.056 | 129.862 | -2.1 |
| MOTHE_c21540 | uroporphyrinogen decarboxylase | 585 | 2.572 | -2.1 |
| MOTHE_c20230 | putative aliphatic sulfonates-binding protein precursor | 21 | 101 | -2.1 |
| MOTHE_c18580 | thiol:disulfide interchange protein DsbD | 82 | 366 | -2.1 |
| MOTHE_c02450 | coenzyme PQQ synthesis protein E | 6.351 | 27.980 | -2.1 |
| MOTHE_c16700 | thiazole synthase | 468 | 2.040 | -2.1 |
| MOTHE_c14060 | iron-sulfur cluster assembly scaffold protein IscU | 158 | 753 | -2.1 |
| MOTHE_c22110 | putative transposase | 1.714 | 7.354 | -2.1 |
| MOTHE_c02060 | PIN domain protein | 203 | 874 | -2.1 |
| MOTHE_c01750 | dTDP-glucose 4,6-dehydratase | 21 | 92 | -2.1 |
| MOTHE_c09460 | histone deacetylase-like amidohydrolase | 1.625 | 6.788 | -2.1 |
| MOTHE_c11350 | HTH-type transcriptional repressor YtrA | 112 | 467 | -2.0 |
| MOTHE_c14050 | transcriptional regulator PerR | 107 | 459 | -2.0 |
| MOTHE_c15770 | flagellar assembly protein H | 147 | 653 | -2.0 |
| MOTHE_c19240 | limonene hydroxylase | 1.450 | 5.888 | -2.0 |
| MOTHE_c21550 | carbohydrate diacid regulator | 188 | 732 | -2.0 |

^1^Mean values of normalized read counts.

**Supplementary table 5: Comparison of the Fdh-Ech from *M. thermoacetica* to the Ech1 and Ech2 from *T. kivui*, the formate hydrogenlyases Hyf and Hyc from *E. coli* and to the Fdh-Mrp-Mbh from** ***T. onnurineus.***

| ***M. thermoacetica* Fdh-Ech** | ***T. kivui* Ech 1** | ***T. kivui* Ech 2** | ***E. coli* Hyf** | ***E. coli* Hyc** | ***T. onnurineus* Fdh-Mrp-Mbh** |
| --- | --- | --- | --- | --- | --- |
| FocA | n.f. | n.f. | FocB 29% | n.f. | FocA 38% |
| FdhB | n.f. | n.f. | n.f. | n.f. | n.f. |
| FdhA | n.f. | n.f. | FdhF 42% | FdhF 42% | Fdh2A 47% |
| HycB | HycB1 35% | HycB2 32% | HyfA 49% | HycB 48% | Fdh2B 34% |
| EchA | Ech1A 31% | Ech2A2 27% | HyfB 49% | HycC 42% | MbhH’ 33% |
| EchB | Ech1B 25% | Ech2B 24% | HyfC 52% | HycD 51% | MbhH’’ 31% |
| EchX | n.f. | n.f. | HyfE 47% | n.f. | n.f. |
| EchY | Ech1A 32% | Ech2A1 29% | HyfF 54% | HycC 28% | MrpH’’’ 30% |
| EchZ | Ech1A 27% | Ech2A1 35% | HyfD 55% | HycC 26% | MbhH 38% |
| EchE | Ech1E 40% | Ech2E 40% | HyfG 63% | HycE 65% | MbhKl 51% |
| EchF | Ech1F 30% | Ech2F 31% | HyfH 50% | HycF 44% | MbhN 37% |
| EchC | Ech1C 37% | Ech2C 49% | HyfI 52% | HycG 54% | MbhJ 34% |

n.f. not found

**Supplementary table 6: Comparison of the NADH dehydrogenase from *M. thermoacetica, E. coli, T. thermophilus, Synechocystis* sp and *T. elongatus*.**

| ***M. thermoacetica*** | ***E. coli*** | ***T. thermophilus*** | ***Synechocystis* sp.**  **PCC 6803** | ***T. elongatus*** |
| --- | --- | --- | --- | --- |
| NqoA | NuoA 28% | Nqo7 41% | NdhC 50% | NdhC 52% |
| NqoB | NuoB 53% | Nqo6 62% | NdhK 55% | NdhK 53% |
| NqoC | NuoC 36% | Nqo5 43% | NdhJ 45% | NdhJ 41% |
| NqoD | NuoD 40% | Nqo4 48% | NdhH 42% | NdhH 44% |
| NqoE (n.f.) | NuoE | Nqo2 | n.f. | n.f. |
| NqoF (n.f.) | NuoF | Nqo1 | n.f. | n.f. |
| NqoG (n.f.) | NuoG | Nqo3 | n.f. | n.f. |
| NqoH | NuoH 42% | Nqo8 42% | NdhA 44% | NdhA 43% |
| NqoI | NuoI 35% | Nqo9 39% | NdhI 35% | NdhI 32% |
| NqoJ | NuoJ 31% | Nqo10 47% | NdhG 46% | NdhG 39% |
| NqoK | NuoK 38% | Nqo11 51% | NdhE 52% | NdhE 52% |
| NqoL | NuoL 41% | Nqo12 46% | NdhF 46% | NdhF 47% |
| NqoM | NuoM 37% | Nqo13 31% | NdhD 37% | NdhD 37% |
| NqoN | NuoN 37% | Nqo14 37% | NdhB 41% | NdhB 41% |
| n.f. | n.f. | n.f. | NdhL | NdhL |
| n.f. | n.f. | n.f. | NdhM | NdhM |
| n.f. | n.f. | n.f. | NdhN | NdhN |
| n.f. | n.f. | n.f. | NdhO | NdhO |
| n.f. | n.f. | n.f. | NdhP | NdhP |
| n.f. | n.f. | n.f. | NdhQ | NdhQ |
| n.f. | n.f. | n.f. | NdhR | NdhR |
| n.f. | n.f. | n.f. | NdhS | NdhS |

n.f. not found

**Supplementary figures**

**Supplementary figure 1. Overview of transcriptional changes in *M. thermoacetica* growing on H_2_+CO_2_.** Depiction of log_2_fold transcriptional changes of genes encoding the WLP, redox balancing enzymes and energy conserving enzymes of cells grown on H_2_+CO_2_ compared to glucose grown cells (*n* =3). Electrons are not balanced, oxidation of MQH_2_ by an electron-bifurcating MTHFR is assumed as well as Fd:quinone oxidoreductase activity of the NADH dehydrogenase. For enzymes containing multiple subunits, the range of expression levels is given.

**Supplementary figure 2**

**Supplementary figure 2. Overview of transcriptional changes in *M. thermoacetica* growing on CO.** Depiction of log_2_fold transcriptional changes of genes encoding the WLP, redox balancing enzymes and energy conserving enzymes of cells grown on CO *vs* glucose grown cells (*n* =3). Electrons are not balanced, oxidation of MQH_2_ by an electron-bifurcating MTHFR is assumed as well as Fd:quinone oxidoreductase activity of the NADH dehydrogenase. For enzymes containing multiple subunits, the range of expression levels is given.
